# Immobilized β-galactosidase BgaC from *Bifidobacterium adolescentis* retains stability and activity during repeated cycles of use

**DOI:** 10.1007/s00253-025-13564-5

**Published:** 2025-07-30

**Authors:** Daniel Mehabie Mulualem, Orla Dwan, Michelle Kilcoyne, Conor O’Byrne, Aoife Boyd

**Affiliations:** 1 Pathogenic Mechanisms Research Group, School of Natural Sciences, University of Galway, Galway, Ireland; 2 Department of Biology (Microbiology Stream), College of Natural & Computational Sciences, University of Gondar, Gondar, Ethiopia; 3 Infectious Disease Section, School of Biological and Chemical Sciences, University of Galway, Galway, Ireland

**Keywords:** BgaC, Immobilization, Entrapment, Calcium alginate, β-Galactosidase, Bifidobacterium

## Abstract

**Abstract:**

β-Galactosidase enzymes catalyze the hydrolysis of terminal non-reducing β-D-galactose residues in β-galactosides. These enzymes are important in producing lactose-free dairy products, reducing the lactose content of whey in dairy products, and for production of galactooligosaccharides (GOS) as prebiotic additives to infant formula. To use β-galactosidases in industrial settings, enzyme immobilization procedures are used to enhance their activity and stability and to minimize enzyme quantities and cost. In this study, recombinant *Bifidobacterium adolescentis* β-galactosidase BgaC was immobilized in calcium alginate and gelatin cross-linked with glutaraldehyde. The kinetic parameters and stability properties of immobilized BgaC were characterized in comparison with free soluble enzyme. The *K*_M_ for immobilized BgaC using ortho-nitrophenyl-β-galactoside (ONPG) was 810 ± 220 μM and the *K*_M_ of free BgaC was 2500 ± 3 μM. The *k*_cat_ and *k*_cat*/*_*K*_M_ of immobilized BgaC were 802 s^−1^ and 990 s^−1^ mM^−1^, respectively, compared to *k*_cat_ and *k*_cat*/*_*K*_M_ values of 209 s^−1^ and 84 s^−1^ mM^−1^, respectively, for free BgaC. Immobilized BgaC β-galactosidase was active at all tested pH (pH 4–10), while the free enzyme had decreased activity at pH < 5.5 and > 8.0. The immobilized enzyme had optimum activity at 40 °C, while the free enzyme was most active at 37 °C. In addition, immobilization enhanced acidic pH and temperature stability compared to the free enzyme. Reutilization of the BgaC beads was assessed and the enzyme maintained 69% activity after 12 rounds of reutilization. Therefore, the enhanced performance properties of immobilized BgaC make it a promising candidate for industrial applications.

**Key points:**

• *Bifidobacterium adolescentis β-galactosidase BgaC was successfully immobilized*

• *Immobilized BgaC has enhanced enzymatic activity and stability and allows recycling*

• *Sustained activity of immobilized BgaC is advantageous for industrial applications*

**Graphical Abstract:**

**Figure:**
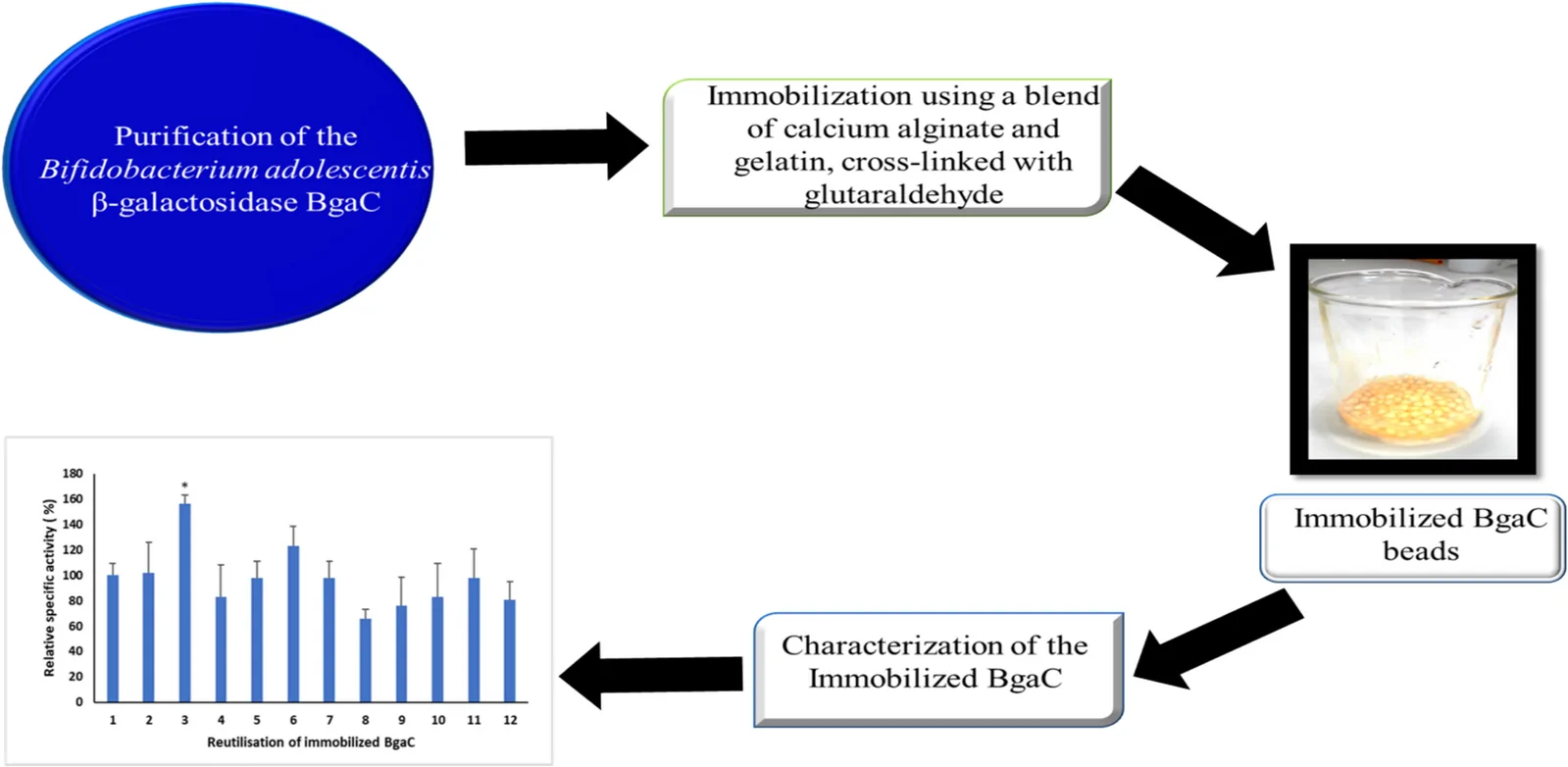

**Supplementary Information:**

The online version contains supplementary material available at 10.1007/s00253-025-13564-5.

## Introduction

Enzymes are routinely used in many industries, including food, textile, detergent, leather, energy, and pharmaceutical production processes (Darbandi et al. 2024). Having desirable properties such as activity in mild reaction conditions and high substrate specificity and productivity, enzymes are frequently preferred to traditional chemical methods (Donzella and Contente 2024). β-Galactosidases (E.C. 3.2.1.23) catalyze the hydrolysis of terminal non-reducing β-D-galactose residues in β-D-galactosides (Drula et al. 2021). They are important in the production of lactose-free dairy products and reducing the lactose content of whey. Moreover, β-galactosidases produce galactooligosaccharides (GOS) via transgalactosylation of lactose, which are important products used as infant formula ingredients. The stability, catalytic activity, and reusability of β-galactosidases are often improved through immobilization procedures for large-scale industrial applications (Sousa et al. 2025).

Since enzymes dissolved in a liquid medium are difficult to retain or recycle, enzymes are immobilized to enable the repeated reutilization of enzymes for industrial processes, which ultimately is cost effective and can potentially increase thermal and pH stability of the enzyme (Mohidem et al. 2023). Immobilization techniques can be broadly categorized into two major classes: carrier-bound and carrier-free immobilization (Cao et al. 2003). In carrier-bound immobilization, the enzymes are bound onto an insoluble carrier using non-covalent adsorption and deposition, ionic interactions, covalent attachment, or entrapment in polymeric gel. Entrapment refers to enzymes enclosed within a carrier matrix without chemically modifying the enzyme (Imam et al. 2021). The most widely used entrapment technique is gel polymerization using agarose, calcium alginate, or a blend of gelatin and calcium alginate (Serey et al. 2021; Yildiz Dalginli and Atakisi 2023). Carrier-free immobilization does not require the presence of a carrier matrix; rather, enzymes are treated with a cross-linking agent, such as glutaraldehyde, to produce insoluble cross-linked enzymes (Bouguerra et al. 2024). A comprehensive review of traditional and modern immobilization techniques and the mechanism of reactions that happen between carrier support and the enzymes is presented in Homaei et al. (2013) and Robescu and Bavaro (2025).

Thoughtful integration of different immobilization techniques has resolved drawbacks that may result from using a single technique. For instance, enzyme leakage in the entrapment technique, which is particularly an issue in calcium alginate beads, has been overcome by cross-linking the enzyme and carrier matrix (Vivek 2024). A further development to overcome enzyme leakage from calcium alginate beads incorporates a blend of gelatin with calcium alginate and cross-linking with glutaraldehyde to produce an insoluble structure which additionally stabilizes the alginate gel (Fadnavis et al. 2003). Critically, this immobilization procedure is cost-effective, can be carried out in a reasonable amount of time, comprises major constituents of the matrix (gelatin and calcium alginate) that are non-toxic, is eco-friendly, and it can be used in the food industry (Mazzocato and Jacquier 2024). Previously, β-galactosidase from *Aspergillus oryzae* (AO.β-Gal) was immobilized in this manner and demonstrated enhanced stability compared to free enzyme with minimal enzyme leakage (Freitas et al. 2012). AO.β-Gal was subsequently authorized for use in food production following its safety evaluation (EFSA Panel on Food Contact Materials et al. 2022).

It was previously demonstrated that the *Bifidobacterium adolescentis* BgaC β-galactosidase efficiently hydrolyzed lactose and carried out transgalactosylation of lactose to produce GOS (Hu et al. 2023; Mulualem et al. 2021; Zhou et al. 2024). BgaC is a 116 kDa protein with closest relatives in *B. ruminantium*, *B. pseudocatenulatum*, *B. tsurumiense*,* B. catenulatum*, and *B. longum* (86%, 75%, 68%, 68%, and 68% amino acid identity, respectively) (Camacho et al. 2009). *Bifidobacterium* β-galactosidases are particularly relevant for infant nutrition because they produce bifidogenic GOS that are selectively utilized by *Bifidobacterium*. The presence of *Bifidobacterium* in the infant gut is associated with numerous host health benefits. Methodologies to immobilize BgaC have not previously been developed and assessed.

In this study, BgaC was immobilized by entrapment in a gelatin and calcium alginate carrier matrix, and cross-linked with glutaraldehyde. The biochemical properties of immobilized BgaC were compared to the soluble enzyme and to immobilized AO.β-Gal. This work demonstrates that immobilized BgaC exhibited enhanced activity and stability and thus can be suitable for industrial applications.

## Material and methods

### Materials

All reagents were purchased from Sigma Aldrich Co. (Dublin, Ireland; now Merck) unless otherwise stated and were of the highest grade available. These included *A. oryzae* β-galactosidase (AO.β-Gal) (cat. no. G5160; ≥ 8.0 units/mg solid), gelatin from bovine skin (Type B powder, cat. no. G6650), sodium alginate (cat. no. 180947), glutaraldehyde solution (grade II, 25% in water), ortho-nitrophenyl-β-galactoside (ONPG), and the glucose-oxidase/peroxidase assay kit.

### Production and purification of BgaC

The gene encoding BgaC β-galactosidase of *B. adolescentis*, which was identified by screening a human fecal metagenome library, had previously been cloned for expression as a His-tagged protein in plasmid pDMg1a (Mulualem et al. 2021). The method of expression and purification of His-BgaC was performed as previously described (Mulualem et al. 2021). In brief, the host *Escherichia coli* T7 Express lacZ^−^ (pDMg1a) was grown at 37 °C to mid-log phase (OD_600_ between 0.4 and 0.7). To induce gene expression of BgaC, 1 mM isopropyl-β-D-thiogalactopyranoside was added, and the culture was incubated for 4 h. Cells were harvested by centrifugation, and the pelleted cells were lysed with Bugbuster protein extraction reagent supplemented with 2 μL/mL lysozyme (Novagen, US). The soluble fraction containing the His-tagged BgaC was purified with Ni^2+^-nitrilotriacetate (Ni^2+^-NTA) affinity chromatography (Thermo Fisher) following the manufacturer’s instructions. The purity of His-BgaC was confirmed by Coomassie-stained SDS-PAGE (Sambrook and Maniatis 1989). The concentration of the purified protein was determined using Bradford assay (Pierce, Ireland) (Bradford 1976).

### Immobilization of BgaC β-galactosidase

Immobilization of purified BgaC was carried out in sodium alginate and gelatin beads cross-linked with glutaraldehyde, essentially as previously described (Freitas et al. 2012). In brief, a 9 mL solution of 6.6% sodium alginate and 4.05% gelatin was heated to 80 °C with continuous stirring until the solution was homogeneous and maintained a jelly-like consistency. The temperature was reduced to below 40 °C, then the solution was put on ice for 60 s, and purified enzyme (1 mL, 430 μg mL^−1^, 37 units mL^−1^) was added to the solution and gently mixed. At room temperature, the enzyme-containing mixture was pumped at a constant speed of 8 rpm using a peristaltic pump (Watson Marlow, United Kingdom) with 0.85 mm diameter tubing into 10 mL 3.64% glutaraldehyde and 0.05 M CaCl_2_ with constant stirring for the formation of immobilized enzyme beads (Freitas et al. 2011). The beads were maintained in the glutaraldehyde and CaCl_2_ solution for 12 h at 4 °C. Afterwards, the beads were washed with 50 mM sodium phosphate buffer, pH 7.0, and maintained in this buffer at 4 °C until used for the enzymatic assays. The concentration of enzyme incorporated in beads was calculated to be 21.5 ng/µL using the initial enzyme amount of 430 μg added to a total bead preparation volume of 20 mL (1 mL enzyme solution, 9 mL alginate and gelatin solution and 10 mL CaCl_2_ and glutaraldehyde solution). AO.β-Gal was immobilized in a similar fashion to that of BgaC, except that the AO.β-Gal immobilized beads were washed and stored in 50 mM sodium acetate buffer, pH 4.5. Storage buffers were selected based on the optimal activity of the enzymes according to manufacturers’ recommendations and previous studies (Mulualem et al. 2021). Negative control beads were also prepared in the same manner without any added enzyme; instead, 1 mL sterile water was added to the 9 mL solution of 6.6% sodium alginate and 4.05% gelatin. The negative control beads were washed with sterile water and stored at 4 °C. The bead preparation was performed three times for the intended experiments. Immobilization activity yield was calculated according to Carević et al. (2018): Activity Yield (%) = (Specific Activity of Immobilized Enzyme/Specific Activity of Free Enzyme) × 100.

### β-Galactosidase specific activity assays

The specific activity of immobilized BgaC was determined using four beads in each reaction. These four beads were equivalent to 250 µL as measured by volume displacement and were calculated to contain 5.4 μg enzyme (equivalent to 21.5 ng/µL). These beads were added to a solution of 250 µL 50 mM sodium phosphate buffer, pH 7.0 supplemented with 10 mM MgCl_2_ and 2 mM ONPG. The reaction was incubated at 37 °C for 30 min. For the free BgaC, the assay was performed in 100 µL containing 50 mM sodium phosphate buffer with 10 mM MgCl_2_, 2 mM ONPG, and 1.08 µg enzyme. The specific activity of the immobilized and free AO.β-Gal was determined in a similar manner except in acetate buffer, pH 4.5, 2 mM ONPG with no MgCl_2_ and at 30 °C. For all assays, the reaction was stopped with an equal volume of 500 mM sodium carbonate, pH 9.0 (250 µL for immobilized protein and 100 µL for free protein), and the absorbance of released *o*-nitrophenol was measured at 420 nm (Biotek microplate spectrophotometer). Specific activity of the enzyme was calculated as µmol *o*-nitrophenol produced/min/mg enzyme (µmol/min/mg).

For assessment of the re-utilization of the immobilized BgaC and AO.β-Gal, the appropriate specific activity assay using ONPG as the substrate was repeated for twelve sequential assays using the same set of beads. The beads were washed four times after each assay with 50 mM sodium phosphate buffer, pH 7.0, for BgaC and 50 mM sodium acetate buffer, pH 4.5, for AO.β-Gal to remove residual *o*-nitrophenol before re-use in the subsequent assay.

The specific activities of the free and immobilized BgaC were also determined using lactose as a substrate in similar assay conditions, but where ONPG was replaced with 5 mM lactose. The free enzyme lactose hydrolysis assay was performed in a 1 mL volume with 4.5 μg enzyme. For the immobilized enzyme, the lactose hydrolysis assay had a final reaction volume of 1.475 mL, with 500 µL beads (measured by volume displacement, containing 0.17 µg/µL of enzyme). The reaction was stopped by deactivating the enzyme at 95 °C for 5 min, and the amount of released glucose was quantified using a commercial glucose-oxidase/peroxidase assay kit according to the manufacturer’s instructions (Khatami et al. 2022).

### Determination of key kinetic parameters

The kinetic parameters of immobilized BgaC were determined in 50 mM sodium phosphate buffer at pH 7.0 as described above using different concentrations of ONPG (0.25–10 mM) as substrate in a time course (0–18 min) enzyme assay. *K*_M_ and *V*_max_ were calculated from the linear part of the progress curve (between 0 and 6 min) of the time course assay followed by non-linear regression fit using the Michaelis–Menten plot using SigmaPlot Software, Enzyme Kinetic Module (Grafiti LLC USA). Additionally, kinetic parameters *k*_cat_ and *k*_cat_/*K*_M_ were determined.

### Effect of pH and temperature on enzyme specific activity and stability

The effect of pH on soluble BgaC activity was assessed in a reaction containing 2 mM ONPG and 1.08 µg purified enzyme. The total volume was adjusted to 100 µL using 50 mM sodium phosphate buffer with 10 mM MgCl_2_ and varying the pH in each reaction from 4 to 10. For immobilized BgaC, the pH in each reaction was varied from 4 to 10 in a final reaction volume of 500 µL containing 250 µL beads (measured by volume displacement, containing 0.021 μg/µL BgaC) in 50 mM sodium phosphate buffer with 10 mM MgCl_2_ and 2 mM ONPG. For pH stability, the free and immobilized BgaC were pre-incubated in 50 mM sodium phosphate buffer adjusted in pH range 4.0–10 for 24 h at 4 °C. The free enzyme preparations and immobilized BgaC (after washing) were then adjusted to pH 7.0. The specific activity was determined using ONPG at pH 7.0 and 37 °C, as previously described.

The effect of temperature on the specific activity of both free and immobilized BgaC was assessed by conducting the specific activity assay with ONPG (as described in Sect. 2.4) over a temperature range of 0–60 °C. To determine the effect of temperature on the stability of the free and immobilized BgaC beads (0.17 µg/µL) and soluble enzyme (0.0227 µg/µL) were pre-incubated at the tested temperature (0–60 °C) in 50 mM sodium phosphate buffer, pH 7.0, for 1 h, and then the specific activity assay was carried out at 37 °C.

### Statistical analysis

All analyses were performed in technical triplicates in three independent experiments. Statistical analyses of the data were conducted using IBM SPSS statistics 20. The results were expressed as the mean ± standard deviation. The statistical significance was determined using ANOVA (F-stat, p-value, df), with Tukey’s honestly significant difference (HSD) post hoc results shown on figures via significance letters/numbers. For the reutilization assay, repeated measures analysis of variance (ANOVA) was used to determine statistical significance. Prior to ANOVA, the assumption of sphericity was assessed using Mauchly’s test of sphericity, which indicated a significant violation of this assumption (W = 0.000, p < 0.001). Consequently, Greenhouse–Geisser correction was applied to the degrees of freedom for all within-subjects effects. Pairwise comparisons were conducted using Bonferroni correction to adjust for multiple comparisons among the reuse rounds. A significance level of α = 0.05 was adopted for all tests.

## Results

### Immobilization and specific activity of recombinant BgaC

The *E. coli* T7 express (pDMg1a) strain which harbors the *B. adolescentis* β-galactosidase BgaC was grown to induce recombinant protein expression, and His-BgaC purification was conducted using Ni^2+^-NTA columns (Mulualem et al. 2021). Purified recombinant BgaC was immobilized with a blend of calcium-alginate and gelatin in the presence of the cross-linker glutaraldehyde. Commercial AO.β-Gal was immobilized in a similar manner for comparative purposes. The average weight of individual beads ranged from 60 to 70 mg, with an average diameter of 4.4 mm and incorporated approximately 1.34 μg enzyme.

The β-galactosidase activity of immobilized BgaC was compared to the activity of free BgaC using ONPG and lactose as substrates (Table 1). Beads generated with water in the absence of enzyme were used as a negative control and, as expected, displayed no β-galactosidase activity. The immobilized BgaC enzyme showed a 132% increase (2.3-fold increase) in specific activity compared to the free enzyme when lactose was used as a substrate and a 41% increase when ONPG was the substrate (Table 1). The specific β-galactosidase activity of immobilized AO.β-Gal with ONPG as substrate was similar to that of immobilized BgaC (Table 1). These data indicated that the β-galactosidase activity of BgaC was not only retained after immobilization but also increased.

The activity yield of immobilized BgaC compared to the free enzyme was 153%. This could be attributed to many factors. The immobilization may stabilize the active form of the enzyme with higher activity compared to the native enzyme as in the case of lipase immobilization (Mateo et al. 2007). Alternatively, immobilization may reduce substrate or product inhibition, as the free enzyme was inhibited by the substrate ONPG at concentrations ≥ 2.5 mM (Mulualem et al. 2021).

### Kinetic parameters of immobilized β-galactosidase BgaC

**Fig. 1:**
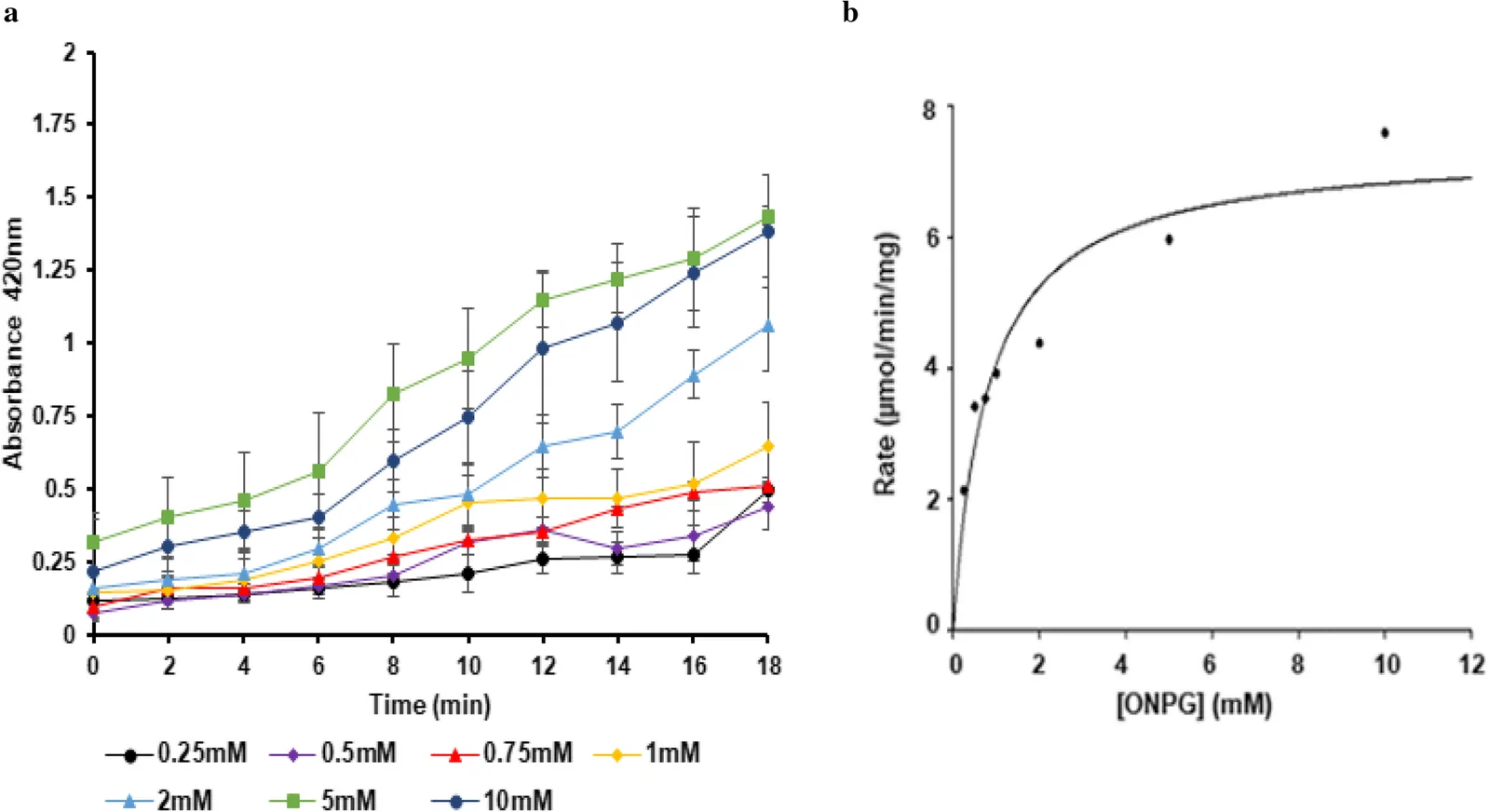
Time course progress curve and non-linear regression fit of immobilized BgaC. Activity was monitored at 2 min intervals over an 18 min period with various concentrations of ONPG (0.25–10 mM). Three independent experiments were conducted, each with three replicates. **a** Time course progress curve of ONPG hydrolysis. Error bars indicate ± 1 standard deviation. **b** Non-linear regression fit model of Michaelis–Menten plot for determination of the kinetic parameters

The kinetic parameters of immobilized BgaC were determined using different concentrations of ONPG (0.25–10 mM) as substrate in a time course (0–18 min) enzyme assay. No substrate or product inhibition was observed at the tested concentrations of ONPG within the progress curve of the assay (Fig. 1a). The *K*_M_ and *V*_max_ of the immobilized enzyme were 0.81 ± 0.22 mM and 7.4 ± 0.63 μmol/min/mg, respectively. The *k*_cat_ and *k*_cat_*/K*_M_ of the immobilized enzyme were 802 s⁻^1^ and 990 s⁻^1^ mM⁻^1^, respectively (Table 1). The *k*_cat_ and *k*_cat_*/K*_M_ values of immobilized BgaC are much higher compared to a previous report for free BgaC (Table 1) which suggests the immobilized BgaC demonstrates high catalytic efficiency. Immobilization of BgaC improved its tolerance to ONPG inhibition, which was previously observed for the free enzyme, where the hydrolytic activity markedly declined at ONPG concentrations higher than 2.5 mM (Mulualem et al. 2021). The *K*_M_ of the immobilized BgaC for ONPG reduced three-fold compared to the *K*_M_ of the free enzyme (Table 1), which indicated that the immobilization increased the affinity of the enzyme towards ONPG. This suggested that this immobilization method did not restrict the movement of substrates and buffer into the carrier matrix, which can happen in many immobilization procedures (Tischer and Kasche 1999).

**Table 1:** Specific β-galactosidase activity, *K*_M_, *V*_max_, *k*_cat_, and *k*_cat_*/K*_M_ for free and immobilized BgaC with ONPG and lactose as substrate

Enzyme preparation	Specific activity (μmol/min/mg)	*K*_M_(mM)	*V*_max_(μmol/min/mg)	*k*_cat_(S^−1^)	*k*_cat_*/K*_M_(S^−1^ mM^−1^)
Immobilized BgaC	52.0 ± 9.0 (ONPG)	0.81 ± 0.22	7.4 ± 0.6	802	990
	89.5 ± 0.3 (Lactose)	n.d. ^a^	n.d.	n.d.	n.d.
Free BgaC	37.0 ± 0.2 (ONPG) ^b^	2.5 ± 3.1^b^	107.0 ± 48.1^b^	209 ^b^	84 ^b^
	38.5 ± 0.1 (Lactose)	3.7 ± 2.0 ^b^	22.0 ± 1.0 ^b^	43 ^b^	12 ^b^
Immobilized AO.β-Gal	61.0 ± 6.8 (ONPG)	n.d.	n.d.	n.d.	n.d.
Free AO.β-Gal	20.2 (ONPG)	n.d.	n.d.	n.d.	n.d.

^a^n.d., not determined

^b^Values determined in previous work ( Mulualem et al. 2021)

### Effect of pH and temperature on specific activity and stability of free and immobilized BgaC

**Fig. 2:**
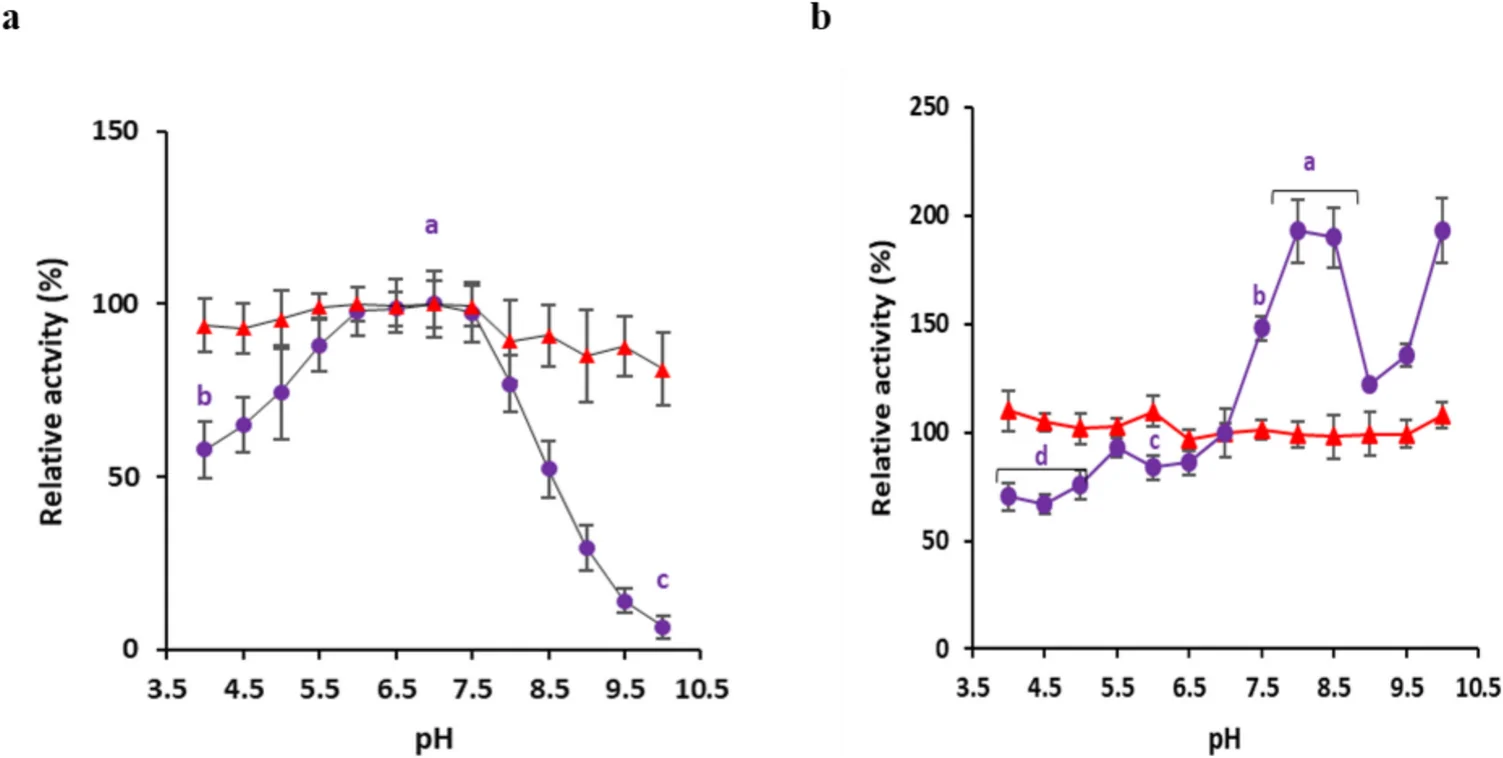
Effect of pH on activity and stability of free and immobilized BgaC. Values represent the mean ± 1 SD of three independent experiments (i.e., three separate batches of immobilized beads). The specific activity value at pH 7 was taken as 100% relative activity. Statistical significance of free or immobilized BgaC at various pH was determined by ANOVA with Tukey’s HSD post hoc test (p < 0.05) (Supplementary Tables 1 and 2). There was no statistical difference in the activity of immobilized BgaC at different pH and therefore statistical significance values are presented only for free BgaC. **a** Effect of pH on the activity of free BgaC (purple circles) and immobilized BgaC (red triangles). Letters (a, b, c) indicate a statistically significant difference between mean enzyme activities at pH 4.0, 7.0, and 10.0, respectively. Statistical values for other pH values are presented in Supplementary Table 1c. **b** pH stability of free BgaC (purple circles) and immobilized BgaC (red triangles). The activity at pH values sharing a common letter is not significantly different. Statistical values for other pH values are presented in Supplementary Table 2c

**Fig. 3:**
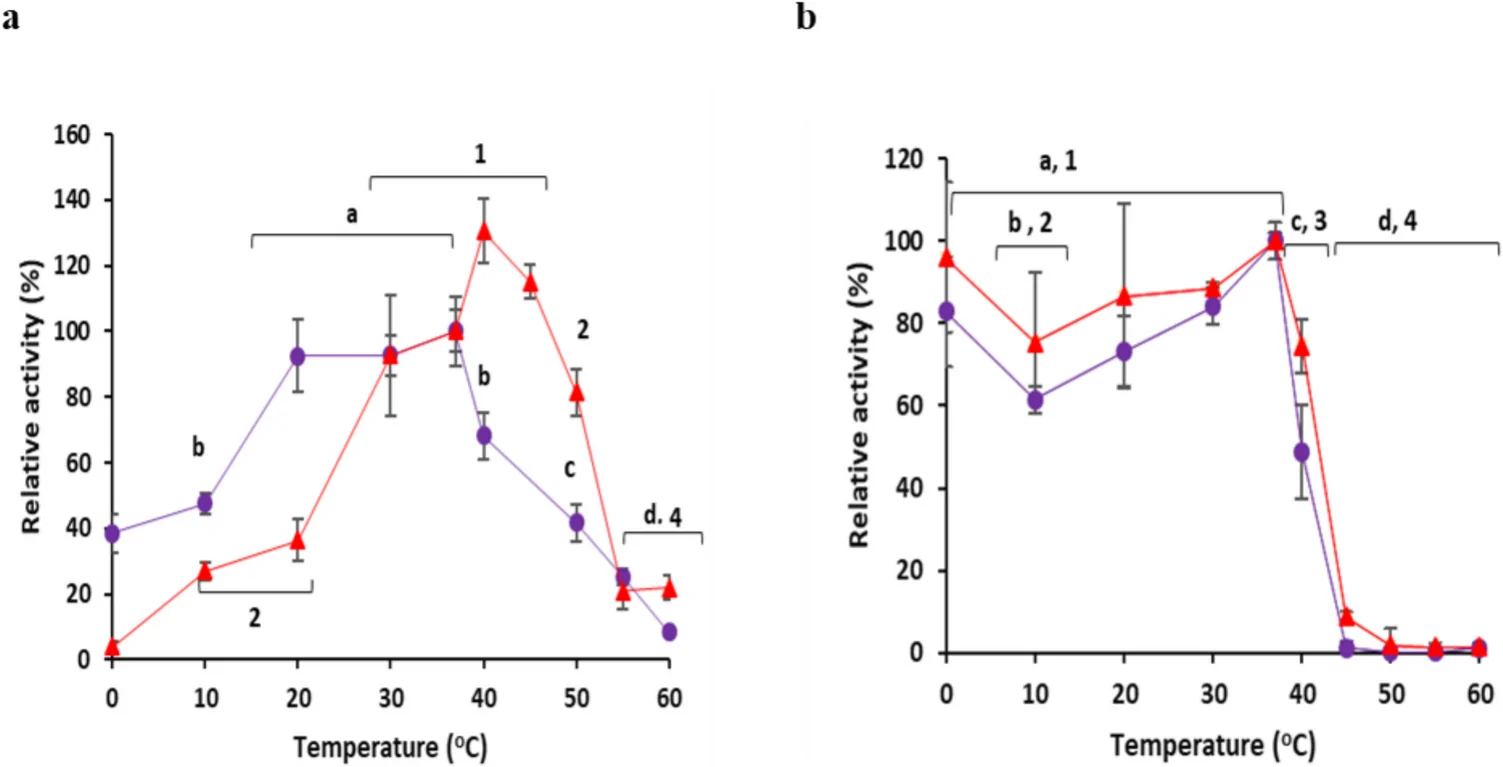
Effect of temperature on activity and stability of free and immobilized BgaC. The values represent the mean ± 1 SD of three independent experiments (three batches of beads for immobilized enzyme). The specific activity value at 37 °C was taken as 100% relative activity. Based on Tukey’s HSD post hoc test, means with different lowercase letters (for free enzyme) or numbers (for immobilized enzyme) are statistically significantly different (p < 0.05), while means sharing the same letter or number are not.** a** Effect of temperature on the activity of free BgaC (purple circles) and immobilized BgaC (red triangles).** b** Effect of temperature on stability of free BgaC (purple circles) and immobilized BgaC (red triangles)

The impact of different pH and temperature ranges on the catalytic activity and stability of free and immobilized BgaC was determined. The specific enzyme activities were examined at pH values between 4 and 10 and temperatures ranging from 0 to 60 °C using ONPG as substrate (Figs. 2 and 3). The optimum pH for β-galactosidase activity of free BgaC was pH 7.0, and the enzyme retained 58% and 52% of its activity at pH 4 and at pH 8.5, respectively (Fig. 2a). However, at pH 10, the activity was nearly abolished. These data were in line with the previous report, although decreased activity at pH 4 and 4.5 was detected here, rather than abolished (Mulualem et al. 2021). On the other hand, immobilized BgaC retained its activity at all the tested pH and retained 93% and 81% activity at pH 4.5 and pH 10, respectively (Fig. 2a). One-Way ANOVA analysis revealed no statistically significant effect of pH on the immobilized enzyme activity, F(12, 26) = 0.19, p = 0.998 (Supplementary Table 1a). This indicates that the immobilized enzyme maintained steady activity across the pH range of 4.0 to 10.0 under the experimental conditions. Therefore, the immobilization procedure extended the pH range for enzyme activity. In contrast, for the free enzyme, One-Way ANOVA revealed a highly statistically significant effect of pH on enzyme activity, F(12,26) = 163.40, p < 0.001, indicating that the mean enzyme activity of the free enzyme differs significantly across at least some of the pH levels tested (Supplementary Table 1b). Further post hoc comparisons using Tukey’s HSD test were performed to identify specific pH levels with significant differences in free enzyme activity (Supplementary Table 1c). The results indicated a distinct optimal pH range, with enzyme activity significantly higher at central pH values compared to acidic (pH 4.0, pH 4.5) and alkaline (pH 9.0, pH 9.5, pH 10.0) conditions. For instance, enzyme activity at pH 7.0 was significantly greater (p < 0.001) than at pH 4.0, 8.5, 9.0, 9.5, and 10.0.

In the case of pH stability, the immobilized BgaC retained activity after 24 h pre-incubation at all tested pH values (Fig. 2b). The free enzyme retained ≥ 66% of residual activity after pre-incubation at acidic pH values (pH 4.0–6.5) and showed increased stability (with some variation detected) following pre-incubation at alkaline pH ranges (pH 7.5–10) compared to pH 7.0 (Fig. 2b). In the previous report, the free enzyme was stable at all tested pH values, with 87% residual activity detected at pH 4 (Mulualem et al. 2021). One-way ANOVA analysis showed no statistically significant effect of different pH conditions on the retained activity of the immobilized enzyme, F(12, 26) = 1.64, p = 0.142 (Supplementary Table 2a). This suggests that the immobilization process conferred robust pH stability to the enzyme, allowing it to maintain consistent activity across the tested pH range (pH 4.0 to 10.0). In contrast, one-way ANOVA conducted for the free enzyme revealed a highly statistically significant effect of pH conditions on its stability, F(12,26) = 57.12, p < 0.001, indicating that the free enzyme loses a significant portion of its activity when exposed to non-optimal pH conditions for extended periods (Supplementary Table 2b). The Tukey’s HSD post hoc test results for the free enzyme pH stability indicate that there were no statistically significant differences in the retained activity of the free enzyme within the acidic pH range (pH 4.0–5.5, with p > 0.05) (Supplementary Table 2c). However, the activity at all acidic pH was significantly lower than at pH where the enzyme exhibited highest stability (e.g., pH 8.0, 8.5 or 10.0) (p < 0.001 for comparisons between pH 4.0–5.5 and pH 8.0, 8.5 or 10.0). In summary, immobilized BgaC showed higher stability than free BgaC at low and high pH ranges, and the immobilization procedure enhanced enzyme stability at acidic pH.

Regarding the effect of temperature, the highest activity for the immobilized BgaC was detected at 40 °C, while the free enzyme had the highest activity at 37 °C (Fig. 3a). Immobilized BgaC retained 81% of its activity at 50 °C, but showed relatively low activity between 0 and 20 °C. The free enzyme retained 68% and 41% of its activity at 40 and 50 °C, respectively. ANOVA analysis revealed a highly significant effect of temperature on the immobilized enzyme, with a large F-statistic [F(9,20) = 7.75] and a very low P-value (p = 7.48 × 10^−5^) (Supplementary Table 3a and 3b). Similarly, free enzyme activity displayed a highly significant effect of temperature, with a larger F-statistic [F(8,18) = 14.50] and an extremely low P-value (p = 2.13 × 10^−6^) (Supplementary Table 3c and 3 d). The higher F-statistic for the free enzyme compared to the immobilized enzyme (F free = 14.50 vs. F immobilized = 7.75) might suggest that the free enzyme is more sensitive to temperature changes than its immobilized counterpart within the tested temperature range.

The maximum temperature stability of both the immobilized and free BgaC was 37 °C, yet 48% and 74% of activity were retained following 1 h pre-incubation at 40 °C by the free and immobilized enzyme, respectively (Fig. 3b). Both the free and immobilized enzymes were inactive after pre-incubation at 45 °C and above; however, ≥ 61% and ≥ 75% residual activity remained following pre-incubation at temperatures of 0–37 °C for the free enzyme and immobilized enzyme, respectively.

ANOVA analysis for the effect of temperature on the stability of immobilized BgaC showed an F-statistic of F(9,20) = 204.06 and a p-value of 1.11 × 10^−17^ (or p < 0.001) (Supplementary Table 4a). In addition, the Tukey’s HSD test revealed that the optimal stability range for immobilized BgaC is 0 °C to 37 °C (Supplementary Table 4b). Most temperatures within this range are not significantly different from each other in terms of stability (e.g., 0 °C vs 20 °C: p-adj = 0.492; 20 °C vs 30 °C: p-adj = 1.0; 30 °C vs 37 °C: p-adj = 0.2582). The ANOVA on the effect of temperature on the stability of free BgaC indicated an F-statistic of F(9,20) = 110.10, with an extremely low p-value of 4.72 × 10 ^−15^ (or p < 0.001) (Supplementary Table 4c). The Tukey’s HSD test revealed that the optimal stability range for free BgaC also extends from 0 °C up to 37 °C (Supplementary Table  4 d). Within this range, temperatures generally do not show statistically significant differences in stability (e.g., 0 °C vs 20 °C: p-adj = 0.7442; 20 °C vs 30 °C: p-adj = 0.5961). In summary, Tukey’s HSD revealed that both free and immobilized BgaC maintain their stability well up to around 37 °C, but then experience a sharp and significant decline starting at 40 °C. However, immobilized BgaC appears to maintain a statistically similar level of stability at 40 °C as it does at 20 °C p-adj (0.2142) or 30 °C with p-adj (0.0921), whereas the free enzyme has already experienced a statistically significant drop at 40 °C compared to those same lower temperatures with p-adj = 0.0076 for 20 °C and p-adj = 0.0001 for 30 °C.

With regard to the effect of storage temperature on the activity of BgaC, storage at 4 °C for 24 h did not affect the activity of the free enzyme or the immobilized BgaC. It was previously reported that free BgaC can be stored at 4 °C for 5 weeks without loss of activity (Mulualem et al. 2021).

### Reutilization of immobilized BgaC and AO.β-Gal

**Fig. 4:**
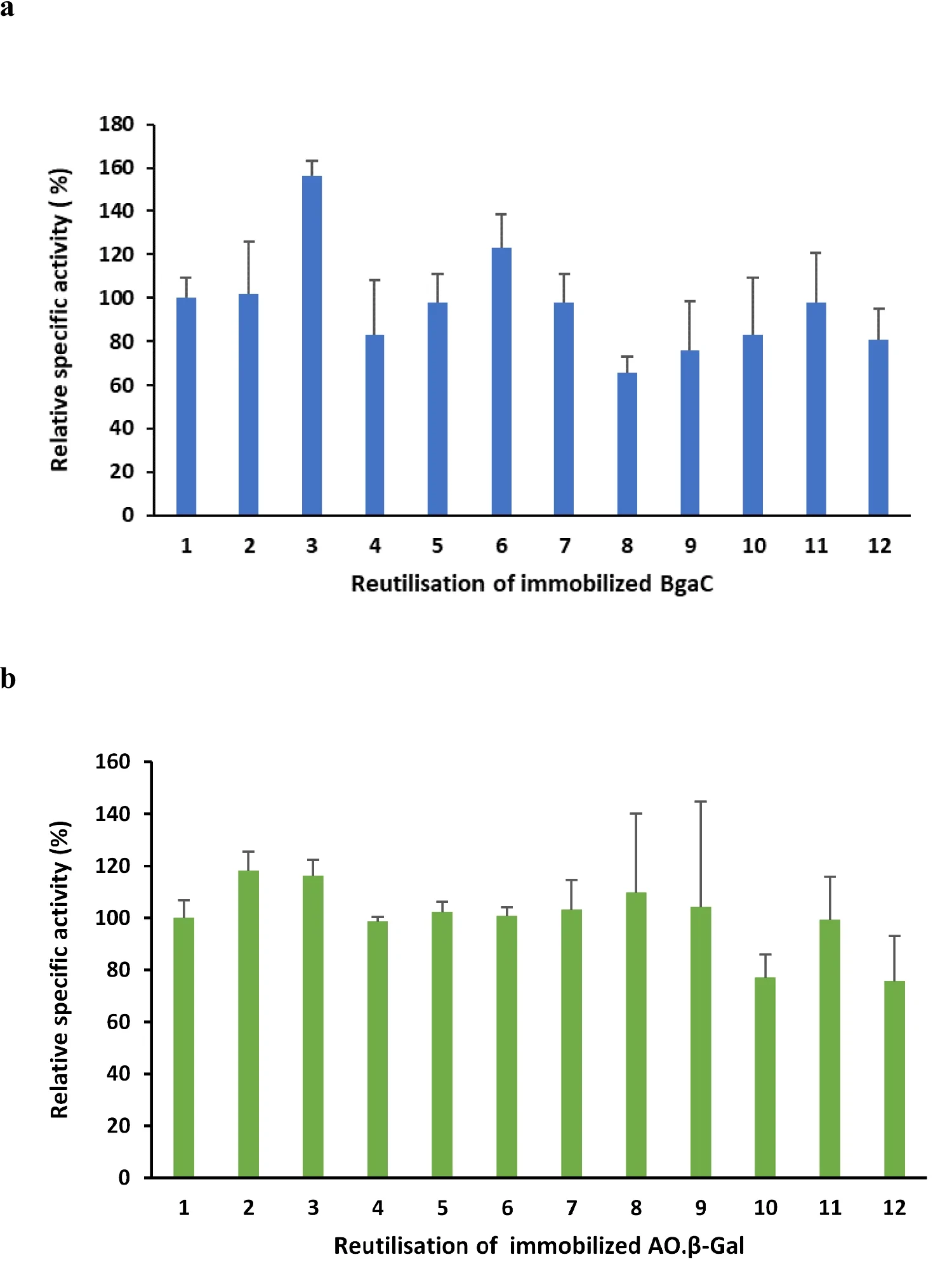
Reutilization of immobilized BgaC and AO.β-Gal. **a** The specific activity of immobilized BgaC and (**b**) the immobilized AO.β-Gal determined for the twelve rounds of reutilization assay using ONPG as a substrate. For both panels the data represent the mean ± SD of relative specific activity values from three independent biological experiments (i.e., three batches of beads for immobilized enzyme). The specific activity value of reuse cycle 1 was taken as 100% relative activity. Statistical significance analysis by repeated measure ANOVA with Greenhouse–Geisser correction showed no statistical differences in enzyme activity throughout the cycles of reutilization. Post hoc Bonferroni-corrected pairwise comparisons revealed that, while the overall effect was not significant, specific activity in Round 3 (mean = 80.88) was significantly higher than in Round 8 (mean = 34.19) (p = 0.002) for immobilized BgaC

The specific activity assay was repeated twelve times sequentially on the same immobilized enzyme beads within the same day. After each assay, the beads were thoroughly washed before reutilization. Both the BgaC and AO.β-Gal showed minimal reduction in activity when the first use was compared to the successive 11 rounds (from 52 ± 9 to 42 ± 15 and from 61 ± 6 to 46 ± 17 μmol/min/mg, respectively) (Fig. 4a and b). There was a trend in declining activity with successive assays overall, although a linear decline in activity was not detected. The activity of the immobilized enzymes showed a slight decrease after the 8th reutilization for BgaC and after the 9th reutilization for AO.β-Gal.

Repeated measures ANOVA was conducted to assess the effect of reuse on specific activity (Supplementary Tables 5a and 5b). For the overall effect of reuse on each immobilized enzyme, Mauchly's Test indicated a significant violation of sphericity (p < 0.001), leading to the application of Greenhouse–Geisser correction. The ANOVA results showed no statistically significant overall effect of reutilization cycle on BgaC specific activity [F(1.243,2.486) = 7.134, p = 0.093]. While not statistically significant, the effect size was large (ηp^2^ = 0.781), suggesting a notable proportion of variance was associated with the reuse rounds, though statistical power might have been a limiting factor given the small sample size (N = 3). Despite the non-significant overall effect, Bonferroni-corrected pairwise comparisons revealed one statistically significant difference: the specific activity in Round 3 (mean, 80.88) was significantly higher than in Round 8 (mean, 34.19) (p = 0.002). All other pairwise comparisons were not statistically significant (p > 0.05). This specific finding would typically be interpreted with caution, acknowledging the non-significant overall test.

The ANOVA results similarly demonstrated no statistically significant overall effect of reuse round on AO.β-gal specific activity [F(1.056,2.112) = 0.857, p = 0.455, ηp^2^ = 0.300]. Tests of within-subjects contrasts revealed no statistically significant linear, quadratic, or higher-order trends. Bonferroni-corrected pairwise comparisons showed no statistically significant differences between any of the reuse rounds (p > 0.05 for all comparisons).

This analysis consistently indicated no significant effect of reuse rounds on the specific activity of immobilized BgaC and immobilized AO.β-gal. It can be concluded that the immobilization procedure using calcium alginate and gelatin cross-linked with glutaraldehyde enabled the retention of enzyme activity for at least 12 successive reutilizations.

## Discussion

Immobilized enzymes have a long history of success for their application in various industries because immobilization can enhance the stability and reusability of enzymes, while maintaining catalytic efficiency (Robescu and Bavaro 2025). In this study, the β-galactosidases BgaC and AO.β-Gal from *B. adolescentis* and *A. oryzae*, respectively, were immobilized in calcium alginate and gelatin beads and cross-linked with glutaraldehyde. Immobilization increased the specific activity of BgaC and increased enzyme affinity for ONPG. The activity yield of immobilized BgaC was 153%, which was relatively higher than the *Kluyveromyces lactis* β-galactosidase immobilized solely with sodium alginate, which had an activity yield (immobilization efficiency) of 66% (Carvalho et al. 2021). The higher yield could be attributed to the mechanical strength and the modification of the encapsulation material to get an optimum microenvironment by attaining an optimal pH, suitable polarity or amphilicity (Maghraby et al. 2023). The procedure also expanded the pH activity range of the enzyme and increased its activity and stability at higher temperatures. Thus, industrially desirable properties of BgaC were enhanced upon immobilization, making it even more suitable for industrial applications.

Immobilized BgaC and AO.β-Gal maintained 69% and 75% activity, respectively, after 12 rounds of reutilization. This is comparable to a previous study in which AO.β-Gal immobilized with entrapment in sodium alginate and gelatin and cross-linking with glutaraldehyde maintained 80% of initial activity after 25 re-uses conducted over 25 days, one use per day (Freitas et al. 2012). Similarly, it was reported that AO.β-Gal immobilized in a blend of calcium alginate and gelatin cross-linked with genipin maintained 90% of its relative activity after 11 reuses in a batch process of lactose hydrolysis (Hackenhaar et al. 2022). Although entrapment of enzymes with calcium alginate beads is simple, economical, and environmentally friendly (Vetrano et al. 2022), it has some drawbacks, such as weak mechanical strength, which can lead to loss of entrapped biomolecules due to swelling in storage or wash buffers (Elnashar et al. 2009). Preliminary experiments entrapped BgaC and AO.β-Gal in only calcium alginate and glutaraldehyde. While the enzymes were successfully immobilized, the beads easily disintegrated after just two rounds of reutilization (data not shown). The addition of gelatin to the entrapment matrix improved bead strength and stability, as shown by the ability to reutilize them in twelve rounds of hydrolysis. Nonetheless, we found that the repeated numerous washing steps of the reutilization protocol led to the carrier matrix of the BgaC beads gradually becoming visibly larger, ultimately resulting in the beads breaking apart after the 12th round. It is possible that absorption of pH 7 wash buffer by the beads during wash steps could have caused bead swelling and some enzyme loss to account for relatively lower activity in reutilization assays at later rounds. The AO.β-Gal immobilized beads maintained integrity and appeared relatively stronger and less fragile compared to the immobilized BgaC beads, which may have been due to washing in acetic acid pH 4.5 buffer.

To maintain the mechanical strength of alginate beads in industrial settings, several key strategies are employed. Vetrano et al. (2023) reported that adding 2 mM Ca^2+^ to the reaction and storage solutions of immobilized horse liver alcohol dehydrogenase in calcium alginate beads maintained the initial structure of the beads, preventing swelling and subsequent breakage. Higher concentrations of Ca^2+^ during gelation create stronger cross-links in the alginate matrix, enhancing the mechanical strength and stability of the beads (Bennacef et al. 2023). Moreover, the pH of the gelation and storage medium also dictates the mechanical strength of calcium-alginate beads, with gel beads being stable at acidic pH but degraded at alkaline pH (Tümtürk et al. 2007). The structure of alginate, (1 → 4)-linked β-D-mannuronic acid and α-L-guluronic acid, accounts for its high stability at acidic pH and low stability and degradation at basic pH (Tumturk et al. 2008). In addition, alginate with high α-L-guluronic acid:β-D-mannuronic acid ratios forms strong and stable beads (Bennacef et al. 2023). The leakage and breakage problem of calcium alginate beads was reduced when AO.β-Gal was immobilized onto alginate/tea waste gel beads via covalent binding through amination with polyethyleneimine followed by activation with glutaraldehyde, which resulted in retention of 99.7% and 72.1% of its initial activity after 15 and 20 consecutive cycles of use, respectively (Abdella and Hassan 2024).

Maintaining catalytic activity at acidic pH is very desirable for industrial applications (Mazzocato and Jacquier 2024). The pH optima of enzymes are known to be affected by the immobilization procedure, which modifies the enzymes via various interactions with the carrier/support, altering the properties of the enzymes (Brena et al. 2013). Moreover, immobilized enzymes exhibit different stability profiles at varying pH levels. The immobilization carrier matrix provides protection from pH-induced denaturation by creating a microenvironment distinct from the reaction mixture (Nguyen and Kim 2017). For example, the pH optimum of immobilized fungal β-galactosidases in an alginate-chitosan blend shifted to a more acidic pH compared to the free enzyme (Katrolia et al. 2019). The Alk-1 xylanase from *Bacillus licheniformis* immobilized within glutaraldehyde-activated calcium alginate beads showed an increase of the optimum pH compared to the free enzyme (from pH 8.0 to 9.0) (Kumar et al. 2017). In agreement with previous reports, the immobilization of BgaC expanded its activity and stability over a wider pH range compared to the free enzyme.

The immobilized BgaC had optimal activity at 40 °C, while the free enzyme had optimal activity at 37 °C. The temperature optima of the free enzyme were 37 °C in the previous report too and retained 60% of activity between 20 and 45 °C (Mulualem et al. 2021). In line with this, AO.β-Gal immobilized using cobalt-alginate cross-linked with glutaraldehyde shifted its temperature optimum to 60 °C, compared to 50 °C for the free enzyme (Ateş and Mehmetoğlu 1997). In addition, immobilization of BgaC enhanced its temperature tolerance compared to the free enzyme as 48% and 74% of activity were retained following 1 h pre-incubation at 40 °C by the free and immobilized enzyme, respectively. Comparable results were previously reported for the free enzyme, where 60% activity was retained after incubation at 40 °C and a decline in activity was detected above 45 °C (Mulualem et al. 2021). A previous study reported that *K. lactis* β-galactosidase immobilized on sodium alginate exhibited enhanced temperature stability, with the highest stability observed at 50 °C for the immobilized enzyme compared to 40 °C for the free enzyme (Carvalho et al. 2021). Similarly, AO.β-Gal immobilized in ConA-layered calcium alginate–starch beads had enhanced activity of lactose hydrolysis and stability to heat and inhibitors compared to the free enzyme (Haider and Husain 2009). The immobilization procedures create a microenvironment that buffers temperature fluctuations and hence protects the structural integrity of enzymes. In addition, immobilization restricts the movement of enzymes under thermal stress, which reduces protein unfolding and aggregation (Khan 2021).

Some immobilization procedures have been suggested to decrease the *K*_M_ of the enzyme as a result of electrostatic interactions between the substrate and the carrier matrix used and diffusion effects (Demirel et al. 2006). In this work, the *K*_M_ of the immobilized BgaC was three-fold lower than the free enzyme (Mulualem et al. 2021), which indicated enhanced affinity of the immobilized enzyme towards the synthetic substrate ONPG. Likewise, immobilized *A. oryzae* amylase on alginate matrix was reported to lower the *K*_M_ value compared to free amylase (from 0.18 mM and 0.15 mM) (Ningsih et al. 2020). Immobilization of AO.β-Gal in activated calcium alginate and tea waste cross-linked with glutaraldehyde reduced its *K*_M_ by 57% compared to the free enzyme (Hassan et al. 2024). In addition, the *k*_cat_ and the *k*_cat_*/K*_M_*values* of immobilized BgaC are greater than free BgaC for hydrolysis of ONPG, which indicates the immobilization procedure enhanced the overall catalytic efficiency of the enzyme. In contrast, the *k*_cat_ of an immobilized β-galactosidase from *K. lactis* via complexation with sodium alginate or Ɩ-carrageenan was not significantly different from the native enzyme (Souza et al. 2018). Immobilization may enhance the activity of some enzymes due to the microenvironment effect (Cao 2005). The specific activity of the immobilized BgaC showed a 2.3-fold increase in lactose hydrolysis compared to the free enzyme. Conversely, it was previously reported that lactose hydrolysis with the free *K. lactis* β-galactosidase was significantly greater for all samples analyzed (bovine whey and fresh whey of bovine, caprine, and buffalo origin) compared to the enzyme immobilized on calcium alginate beads (Argenta et al. 2021).

The novelty of this work is the development of immobilization methodology for BgaC β-galactosidase of *B. adolescentis* and characterization of the biochemical properties of the immobilized BgaC compared to free BgaC. This is the first report of the immobilization of the BgaC enzyme, the first report of the biochemical properties of immobilized BgaC, and the first report of multiple active reutilizations of BgaC-encapsulated beads. In conclusion, the immobilization of BgaC in calcium alginate-gelatin beads cross-linked with glutaraldehyde shifted the optimal temperature for BgaC enzymatic activity to 40 °C, compared to 37 °C for free enzyme. Moreover, the pH range for activity and stability of immobilized BgaC was expanded compared to the free enzyme. Immobilized BgaC was successfully reused 12 successive times for ONPG hydrolysis, suggesting the suitability of the encapsulated BgaC beads for application in fixed bed reactors. This effective enzyme reutilization indicates that this BgaC immobilization procedure could be suitable for continuous industrial processing applications, such as the production of lactose-free dairy products and manufacture of lactose-based prebiotics. In addition, this immobilized BgaC could be utilized for bioethanol production by processing dairy industry by-products, such as whey permeate. These by-products are rich in lactose and could be converted into fermentable sugars by BgaC, which can subsequently be used by various yeast strains for bioethanol production (Murari et al. 2019). Therefore, we recommend further studies be carried out to increase long term stability of the bead matrix and to establish scale up parameters of the immobilization procedure of BgaC for the operational conditions in industry settings.

## Supplementary Information

Below is the link to the electronic supplementary material.

Supplementary Material 1 (DOCX 236 KB)

## Data Availability

Data and material for this article are available upon request.
