# Supplementary material for: Immobilized β-galactosidase BgaC from Bifidobacterium adolescentis retains stability and activity during repeated cycles of use
Source: Appl Microbiol Biotechnol. 2025 Jul 30;109(1):174. doi: 10.1007/s00253-025-13564-5 (PMC12310786; doi:10.1007/s00253-025-13564-5)
Supplement: Supplementary file 1 — Supplementary Material 1 (DOCX 236 KB) [file 253_2025_13564_MOESM1_ESM.docx]

| ANOVA |  |  |  |  |  |  |
| --- | --- | --- | --- | --- | --- | --- |
| *Source of Variation* | *SS* | *df* | *MS* | *F* | *P-value* | *F crit* |
| Between Groups | 0.067773013 | 12 | 0.00564775 | 0.19464192 | 0.997563 | 2.147926 |
| Within Groups | 0.754418827 | 26 | 0.02901611 |  |  |  |
|  |  |  |  |  |  |  |
| Total | 0.82219184 | 38 |  |  |  |  |

**Supplementary Table 1a: A one-way ANOVA for effect of pH on the activity of immobilized BgaC**

**Supplementary Table 1b: A one-way ANOVA for the effect of pH on the activity of free BgaC**

| ANOVA |  |  |  |  |  |  |
| --- | --- | --- | --- | --- | --- | --- |
| *Source of Variation* | *SS* | *df* | *MS* | *F* | *P-value* | *F crit* |
| Between Groups | 16.6053729 | 12 | 1.383781 | 163.3975 | 2.66E-21 | 2.147926 |
| Within Groups | 0.22018889 | 26 | 0.008469 |  |  |  |
|  |  |  |  |  |  |  |
| Total | 16.8255618 | 38 |  |  |  |  |

**Supplementary Table 1c: Tukey's Honestly Significant Difference (HSD) Post-Hoc Test Results for the effect of pH on activity of Free BgaC**

| **Multiple Comparisons** | | | | | | |
| --- | --- | --- | --- | --- | --- | --- |
| Dependent Variable: 420nm  Tukey HSD | | | | | | |
| (I) 4-10 | (J) 4-10 | Mean Difference (I-J) | Std. Error | Sig. | 95% Confidence Interval | |
|  |  |  |  |  | Lower Bound | Upper Bound |
| 4.0 | 4.5 | -.129777777667 | .075138997453 | .866 | -.40283570767 | .14328015234 |
|  | 5.0 | -.326777777667^*^ | .075138997453 | .009 | -.59983570767 | -.05371984766 |
|  | 5.5 | -.605777777667^*^ | .075138997453 | .000 | -.87883570767 | -.33271984766 |
|  | 6.0 | -.838222222333^*^ | .075138997453 | .000 | -1.11128015234 | -.56516429233 |
|  | 6.5 | -.834444444333^*^ | .075138997453 | .000 | -1.10750237434 | -.56138651433 |
|  | 7.0 | -.864555555333^*^ | .075138997453 | .000 | -1.13761348534 | -.59149762533 |
|  | 7.5 | -.816000000000^*^ | .075138997453 | .000 | -1.08905793000 | -.54294207000 |
|  | 8.0 | -.384555555333^*^ | .075138997453 | .001 | -.65761348534 | -.11149762533 |
|  | 8.5 | .133111111333 | .075138997453 | .846 | -.13994681867 | .40616904134 |
|  | 9.0 | .605000000000^*^ | .075138997453 | .000 | .33194207000 | .87805793000 |
|  | 9.5 | .924111111333^*^ | .075138997453 | .000 | .65105318133 | 1.19716904134 |
|  | 10.0 | 1.082444444667^*^ | .075138997453 | .000 | .80938651466 | 1.35550237467 |
| 4.5 | 4.0 | .129777777667 | .075138997453 | .866 | -.14328015234 | .40283570767 |
|  | 5.0 | -.197000000000 | .075138997453 | .346 | -.47005793000 | .07605793000 |
|  | 5.5 | -.476000000000^*^ | .075138997453 | .000 | -.74905793000 | -.20294207000 |
|  | 6.0 | -.708444444667^*^ | .075138997453 | .000 | -.98150237467 | -.43538651466 |
|  | 6.5 | -.704666666667^*^ | .075138997453 | .000 | -.97772459667 | -.43160873666 |
|  | 7.0 | -.734777777667^*^ | .075138997453 | .000 | -1.00783570767 | -.46171984766 |
|  | 7.5 | -.686222222333^*^ | .075138997453 | .000 | -.95928015234 | -.41316429233 |
|  | 8.0 | -.254777777667 | .075138997453 | .084 | -.52783570767 | .01828015234 |
|  | 8.5 | .262888889000 | .075138997453 | .067 | -.01016904100 | .53594681900 |
|  | 9.0 | .734777777667^*^ | .075138997453 | .000 | .46171984766 | 1.00783570767 |
|  | 9.5 | 1.053888889000^*^ | .075138997453 | .000 | .78083095900 | 1.32694681900 |
|  | 10.0 | 1.212222222333^*^ | .075138997453 | .000 | .93916429233 | 1.48528015234 |
| 5.0 | 4.0 | .326777777667^*^ | .075138997453 | .009 | .05371984766 | .59983570767 |
|  | 4.5 | .197000000000 | .075138997453 | .346 | -.07605793000 | .47005793000 |
|  | 5.5 | -.279000000000^*^ | .075138997453 | .042 | -.55205793000 | -.00594207000 |
|  | 6.0 | -.511444444667^*^ | .075138997453 | .000 | -.78450237467 | -.23838651466 |
|  | 6.5 | -.507666666667^*^ | .075138997453 | .000 | -.78072459667 | -.23460873666 |
|  | 7.0 | -.537777777667^*^ | .075138997453 | .000 | -.81083570767 | -.26471984766 |
|  | 7.5 | -.489222222333^*^ | .075138997453 | .000 | -.76228015234 | -.21616429233 |
|  | 8.0 | -.057777777667 | .075138997453 | 1.000 | -.33083570767 | .21528015234 |
|  | 8.5 | .459888889000^*^ | .075138997453 | .000 | .18683095900 | .73294681900 |
|  | 9.0 | .931777777667^*^ | .075138997453 | .000 | .65871984766 | 1.20483570767 |
|  | 9.5 | 1.250888889000^*^ | .075138997453 | .000 | .97783095900 | 1.52394681900 |
|  | 10.0 | 1.409222222333^*^ | .075138997453 | .000 | 1.13616429233 | 1.68228015234 |
| 5.5 | 4.0 | .605777777667^*^ | .075138997453 | .000 | .33271984766 | .87883570767 |
|  | 4.5 | .476000000000^*^ | .075138997453 | .000 | .20294207000 | .74905793000 |
|  | 5.0 | .279000000000^*^ | .075138997453 | .042 | .00594207000 | .55205793000 |
|  | 6.0 | -.232444444667 | .075138997453 | .153 | -.50550237467 | .04061348534 |
|  | 6.5 | -.228666666667 | .075138997453 | .169 | -.50172459667 | .04439126334 |
|  | 7.0 | -.258777777667 | .075138997453 | .075 | -.53183570767 | .01428015234 |
|  | 7.5 | -.210222222333 | .075138997453 | .261 | -.48328015234 | .06283570767 |
|  | 8.0 | .221222222333 | .075138997453 | .202 | -.05183570767 | .49428015234 |
|  | 8.5 | .738888889000^*^ | .075138997453 | .000 | .46583095900 | 1.01194681900 |
|  | 9.0 | 1.210777777667^*^ | .075138997453 | .000 | .93771984766 | 1.48383570767 |
|  | 9.5 | 1.529888889000^*^ | .075138997453 | .000 | 1.25683095900 | 1.80294681900 |
|  | 10.0 | 1.688222222333^*^ | .075138997453 | .000 | 1.41516429233 | 1.96128015234 |
| 6.0 | 4.0 | .838222222333^*^ | .075138997453 | .000 | .56516429233 | 1.11128015234 |
|  | 4.5 | .708444444667^*^ | .075138997453 | .000 | .43538651466 | .98150237467 |
|  | 5.0 | .511444444667^*^ | .075138997453 | .000 | .23838651466 | .78450237467 |
|  | 5.5 | .232444444667 | .075138997453 | .153 | -.04061348534 | .50550237467 |
|  | 6.5 | .003777778000 | .075138997453 | 1.000 | -.26928015200 | .27683570800 |
|  | 7.0 | -.026333333000 | .075138997453 | 1.000 | -.29939126300 | .24672459700 |
|  | 7.5 | .022222222333 | .075138997453 | 1.000 | -.25083570767 | .29528015234 |
|  | 8.0 | .453666667000^*^ | .075138997453 | .000 | .18060873700 | .72672459700 |
|  | 8.5 | .971333333667^*^ | .075138997453 | .000 | .69827540366 | 1.24439126367 |
|  | 9.0 | 1.443222222333^*^ | .075138997453 | .000 | 1.17016429233 | 1.71628015234 |
|  | 9.5 | 1.762333333667^*^ | .075138997453 | .000 | 1.48927540366 | 2.03539126367 |
|  | 10.0 | 1.920666667000^*^ | .075138997453 | .000 | 1.64760873700 | 2.19372459700 |
| 6.5 | 4.0 | .834444444333^*^ | .075138997453 | .000 | .56138651433 | 1.10750237434 |
|  | 4.5 | .704666666667^*^ | .075138997453 | .000 | .43160873666 | .97772459667 |
|  | 5.0 | .507666666667^*^ | .075138997453 | .000 | .23460873666 | .78072459667 |
|  | 5.5 | .228666666667 | .075138997453 | .169 | -.04439126334 | .50172459667 |
|  | 6.0 | -.003777778000 | .075138997453 | 1.000 | -.27683570800 | .26928015200 |
|  | 7.0 | -.030111111000 | .075138997453 | 1.000 | -.30316904100 | .24294681900 |
|  | 7.5 | .018444444333 | .075138997453 | 1.000 | -.25461348567 | .29150237434 |
|  | 8.0 | .449888889000^*^ | .075138997453 | .000 | .17683095900 | .72294681900 |
|  | 8.5 | .967555555667^*^ | .075138997453 | .000 | .69449762566 | 1.24061348567 |
|  | 9.0 | 1.439444444333^*^ | .075138997453 | .000 | 1.16638651433 | 1.71250237434 |
|  | 9.5 | 1.758555555667^*^ | .075138997453 | .000 | 1.48549762566 | 2.03161348567 |
|  | 10.0 | 1.916888889000^*^ | .075138997453 | .000 | 1.64383095900 | 2.18994681900 |
| 7.0 | 4.0 | .864555555333^*^ | .075138997453 | .000 | .59149762533 | 1.13761348534 |
|  | 4.5 | .734777777667^*^ | .075138997453 | .000 | .46171984766 | 1.00783570767 |
|  | 5.0 | .537777777667^*^ | .075138997453 | .000 | .26471984766 | .81083570767 |
|  | 5.5 | .258777777667 | .075138997453 | .075 | -.01428015234 | .53183570767 |
|  | 6.0 | .026333333000 | .075138997453 | 1.000 | -.24672459700 | .29939126300 |
|  | 6.5 | .030111111000 | .075138997453 | 1.000 | -.24294681900 | .30316904100 |
|  | 7.5 | .048555555333 | .075138997453 | 1.000 | -.22450237467 | .32161348534 |
|  | 8.0 | .480000000000^*^ | .075138997453 | .000 | .20694207000 | .75305793000 |
|  | 8.5 | .997666666667^*^ | .075138997453 | .000 | .72460873666 | 1.27072459667 |
|  | 9.0 | 1.469555555333^*^ | .075138997453 | .000 | 1.19649762533 | 1.74261348534 |
|  | 9.5 | 1.788666666667^*^ | .075138997453 | .000 | 1.51560873666 | 2.06172459667 |
|  | 10.0 | 1.947000000000^*^ | .075138997453 | .000 | 1.67394207000 | 2.22005793000 |
| 7.5 | 4.0 | .816000000000^*^ | .075138997453 | .000 | .54294207000 | 1.08905793000 |
|  | 4.5 | .686222222333^*^ | .075138997453 | .000 | .41316429233 | .95928015234 |
|  | 5.0 | .489222222333^*^ | .075138997453 | .000 | .21616429233 | .76228015234 |
|  | 5.5 | .210222222333 | .075138997453 | .261 | -.06283570767 | .48328015234 |
|  | 6.0 | -.022222222333 | .075138997453 | 1.000 | -.29528015234 | .25083570767 |
|  | 6.5 | -.018444444333 | .075138997453 | 1.000 | -.29150237434 | .25461348567 |
|  | 7.0 | -.048555555333 | .075138997453 | 1.000 | -.32161348534 | .22450237467 |
|  | 8.0 | .431444444667^*^ | .075138997453 | .000 | .15838651466 | .70450237467 |
|  | 8.5 | .949111111333^*^ | .075138997453 | .000 | .67605318133 | 1.22216904134 |
|  | 9.0 | 1.421000000000^*^ | .075138997453 | .000 | 1.14794207000 | 1.69405793000 |
|  | 9.5 | 1.740111111333^*^ | .075138997453 | .000 | 1.46705318133 | 2.01316904134 |
|  | 10.0 | 1.898444444667^*^ | .075138997453 | .000 | 1.62538651466 | 2.17150237467 |
| 8.0 | 4.0 | .384555555333^*^ | .075138997453 | .001 | .11149762533 | .65761348534 |
|  | 4.5 | .254777777667 | .075138997453 | .084 | -.01828015234 | .52783570767 |
|  | 5.0 | .057777777667 | .075138997453 | 1.000 | -.21528015234 | .33083570767 |
|  | 5.5 | -.221222222333 | .075138997453 | .202 | -.49428015234 | .05183570767 |
|  | 6.0 | -.453666667000^*^ | .075138997453 | .000 | -.72672459700 | -.18060873700 |
|  | 6.5 | -.449888889000^*^ | .075138997453 | .000 | -.72294681900 | -.17683095900 |
|  | 7.0 | -.480000000000^*^ | .075138997453 | .000 | -.75305793000 | -.20694207000 |
|  | 7.5 | -.431444444667^*^ | .075138997453 | .000 | -.70450237467 | -.15838651466 |
|  | 8.5 | .517666666667^*^ | .075138997453 | .000 | .24460873666 | .79072459667 |
|  | 9.0 | .989555555333^*^ | .075138997453 | .000 | .71649762533 | 1.26261348534 |
|  | 9.5 | 1.308666666667^*^ | .075138997453 | .000 | 1.03560873666 | 1.58172459667 |
|  | 10.0 | 1.467000000000^*^ | .075138997453 | .000 | 1.19394207000 | 1.74005793000 |
| 8.5 | 4.0 | -.133111111333 | .075138997453 | .846 | -.40616904134 | .13994681867 |
|  | 4.5 | -.262888889000 | .075138997453 | .067 | -.53594681900 | .01016904100 |
|  | 5.0 | -.459888889000^*^ | .075138997453 | .000 | -.73294681900 | -.18683095900 |
|  | 5.5 | -.738888889000^*^ | .075138997453 | .000 | -1.01194681900 | -.46583095900 |
|  | 6.0 | -.971333333667^*^ | .075138997453 | .000 | -1.24439126367 | -.69827540366 |
|  | 6.5 | -.967555555667^*^ | .075138997453 | .000 | -1.24061348567 | -.69449762566 |
|  | 7.0 | -.997666666667^*^ | .075138997453 | .000 | -1.27072459667 | -.72460873666 |
|  | 7.5 | -.949111111333^*^ | .075138997453 | .000 | -1.22216904134 | -.67605318133 |
|  | 8.0 | -.517666666667^*^ | .075138997453 | .000 | -.79072459667 | -.24460873666 |
|  | 9.0 | .471888888667^*^ | .075138997453 | .000 | .19883095866 | .74494681867 |
|  | 9.5 | .791000000000^*^ | .075138997453 | .000 | .51794207000 | 1.06405793000 |
|  | 10.0 | .949333333333^*^ | .075138997453 | .000 | .67627540333 | 1.22239126334 |
| 9.0 | 4.0 | -.605000000000^*^ | .075138997453 | .000 | -.87805793000 | -.33194207000 |
|  | 4.5 | -.734777777667^*^ | .075138997453 | .000 | -1.00783570767 | -.46171984766 |
|  | 5.0 | -.931777777667^*^ | .075138997453 | .000 | -1.20483570767 | -.65871984766 |
|  | 5.5 | -1.210777777667^*^ | .075138997453 | .000 | -1.48383570767 | -.93771984766 |
|  | 6.0 | -1.443222222333^*^ | .075138997453 | .000 | -1.71628015234 | -1.17016429233 |
|  | 6.5 | -1.439444444333^*^ | .075138997453 | .000 | -1.71250237434 | -1.16638651433 |
|  | 7.0 | -1.469555555333^*^ | .075138997453 | .000 | -1.74261348534 | -1.19649762533 |
|  | 7.5 | -1.421000000000^*^ | .075138997453 | .000 | -1.69405793000 | -1.14794207000 |
|  | 8.0 | -.989555555333^*^ | .075138997453 | .000 | -1.26261348534 | -.71649762533 |
|  | 8.5 | -.471888888667^*^ | .075138997453 | .000 | -.74494681867 | -.19883095866 |
|  | 9.5 | .319111111333^*^ | .075138997453 | .012 | .04605318133 | .59216904134 |
|  | 10.0 | .477444444667^*^ | .075138997453 | .000 | .20438651466 | .75050237467 |
| 9.5 | 4.0 | -.924111111333^*^ | .075138997453 | .000 | -1.19716904134 | -.65105318133 |
|  | 4.5 | -1.053888889000^*^ | .075138997453 | .000 | -1.32694681900 | -.78083095900 |
|  | 5.0 | -1.250888889000^*^ | .075138997453 | .000 | -1.52394681900 | -.97783095900 |
|  | 5.5 | -1.529888889000^*^ | .075138997453 | .000 | -1.80294681900 | -1.25683095900 |
|  | 6.0 | -1.762333333667^*^ | .075138997453 | .000 | -2.03539126367 | -1.48927540366 |
|  | 6.5 | -1.758555555667^*^ | .075138997453 | .000 | -2.03161348567 | -1.48549762566 |
|  | 7.0 | -1.788666666667^*^ | .075138997453 | .000 | -2.06172459667 | -1.51560873666 |
|  | 7.5 | -1.740111111333^*^ | .075138997453 | .000 | -2.01316904134 | -1.46705318133 |
|  | 8.0 | -1.308666666667^*^ | .075138997453 | .000 | -1.58172459667 | -1.03560873666 |
|  | 8.5 | -.791000000000^*^ | .075138997453 | .000 | -1.06405793000 | -.51794207000 |
|  | 9.0 | -.319111111333^*^ | .075138997453 | .012 | -.59216904134 | -.04605318133 |
|  | 10.0 | .158333333333 | .075138997453 | .658 | -.11472459667 | .43139126334 |
| 10.0 | 4.0 | -1.082444444667^*^ | .075138997453 | .000 | -1.35550237467 | -.80938651466 |
|  | 4.5 | -1.212222222333^*^ | .075138997453 | .000 | -1.48528015234 | -.93916429233 |
|  | 5.0 | -1.409222222333^*^ | .075138997453 | .000 | -1.68228015234 | -1.13616429233 |
|  | 5.5 | -1.688222222333^*^ | .075138997453 | .000 | -1.96128015234 | -1.41516429233 |
|  | 6.0 | -1.920666667000^*^ | .075138997453 | .000 | -2.19372459700 | -1.64760873700 |
|  | 6.5 | -1.916888889000^*^ | .075138997453 | .000 | -2.18994681900 | -1.64383095900 |
|  | 7.0 | -1.947000000000^*^ | .075138997453 | .000 | -2.22005793000 | -1.67394207000 |
|  | 7.5 | -1.898444444667^*^ | .075138997453 | .000 | -2.17150237467 | -1.62538651466 |
|  | 8.0 | -1.467000000000^*^ | .075138997453 | .000 | -1.74005793000 | -1.19394207000 |
|  | 8.5 | -.949333333333^*^ | .075138997453 | .000 | -1.22239126334 | -.67627540333 |
|  | 9.0 | -.477444444667^*^ | .075138997453 | .000 | -.75050237467 | -.20438651466 |
|  | 9.5 | -.158333333333 | .075138997453 | .658 | -.43139126334 | .11472459667 |
| *. The mean difference is significant at the 0.05 level. | | | | | | |

**Supplementary Table 2a: A one-way ANOVA for the effect of pH on the stability of immobilized BgaC**

| ANOVA |  |  |  |  |  |  |
| --- | --- | --- | --- | --- | --- | --- |
| *Source of Variation* | *SS* | *df* | *MS* | *F* | *P-value* | *F crit* |
| Between Groups | 0.105947 | 12 | 0.008829 | 1.638016 | 0.141651 | 2.147926 |
| Within Groups | 0.140141 | 26 | 0.00539 |  |  |  |
|  |  |  |  |  |  |  |
| Total | 0.246088 | 38 |  |  |  |  |

**Supplementary Table 2b: A one-way ANOVA for the effect of pH on the stability of free BgaC**

| ANOVA |  |  |  |  |  |  |
| --- | --- | --- | --- | --- | --- | --- |
| *Source of Variation* | *SS* | *df* | *MS* | *F* | *P-value* | *F crit* |
| Between Groups | 8.279892 | 12 | 0.689991 | 57.11592 | 1.5E-15 | 2.147926 |
| Within Groups | 0.314094 | 26 | 0.012081 |  |  |  |
|  |  |  |  |  |  |  |
| Total | 8.593986 | 38 |  |  |  |  |

**Supplementary Table 2c: Tukey's Honestly Significant Difference (HSD) Post-Hoc Test Results for the effect of pH on stability of Free BgaC**

| **Multiple Comparisons** | | | | | | |
| --- | --- | --- | --- | --- | --- | --- |
| Dependent Variable: 420nm  Tukey HSD | | | | | | |
| (I) 4-10 | (J) 4-10 | Mean Difference (I-J) | Std. Error | Sig. | 95% Confidence Interval | |
|  |  |  |  |  | Lower Bound | Upper Bound |
| 4.0 | 4.5 | .036000000000 | .089742366322 | 1.000 | -.29012711923 | .36212711923 |
|  | 5.0 | -.053777778000 | .089742366322 | 1.000 | -.37990489723 | .27234934123 |
|  | 5.5 | -.228888889000 | .089742366322 | .385 | -.55501600823 | .09723823023 |
|  | 6.0 | -.144888889000 | .089742366322 | .910 | -.47101600823 | .18123823023 |
|  | 6.5 | -.161888889000 | .089742366322 | .831 | -.48801600823 | .16423823023 |
|  | 7.0 | -.302888888667 | .089742366322 | .087 | -.62901600790 | .02323823056 |
|  | 7.5 | -.785888889000^*^ | .089742366322 | .000 | -1.11201600823 | -.45976176977 |
|  | 8.0 | -1.230444444333^*^ | .089742366322 | .000 | -1.55657156356 | -.90431732510 |
|  | 8.5 | -1.201222222000^*^ | .089742366322 | .000 | -1.52734934123 | -.87509510277 |
|  | 9.0 | -.529666666667^*^ | .089742366322 | .000 | -.85579378590 | -.20353954744 |
|  | 9.5 | -.664555555333^*^ | .089742366322 | .000 | -.99068267456 | -.33842843610 |
|  | 10.0 | -1.232000000000^*^ | .089742366322 | .000 | -1.55812711923 | -.90587288077 |
| 4.5 | 4.0 | -.036000000000 | .089742366322 | 1.000 | -.36212711923 | .29012711923 |
|  | 5.0 | -.089777778000 | .089742366322 | .998 | -.41590489723 | .23634934123 |
|  | 5.5 | -.264888889000 | .089742366322 | .200 | -.59101600823 | .06123823023 |
|  | 6.0 | -.180888889000 | .089742366322 | .715 | -.50701600823 | .14523823023 |
|  | 6.5 | -.197888889000 | .089742366322 | .596 | -.52401600823 | .12823823023 |
|  | 7.0 | -.338888888667^*^ | .089742366322 | .036 | -.66501600790 | -.01276176944 |
|  | 7.5 | -.821888889000^*^ | .089742366322 | .000 | -1.14801600823 | -.49576176977 |
|  | 8.0 | -1.266444444333^*^ | .089742366322 | .000 | -1.59257156356 | -.94031732510 |
|  | 8.5 | -1.237222222000^*^ | .089742366322 | .000 | -1.56334934123 | -.91109510277 |
|  | 9.0 | -.565666666667^*^ | .089742366322 | .000 | -.89179378590 | -.23953954744 |
|  | 9.5 | -.700555555333^*^ | .089742366322 | .000 | -1.02668267456 | -.37442843610 |
|  | 10.0 | -1.268000000000^*^ | .089742366322 | .000 | -1.59412711923 | -.94187288077 |
| 5.0 | 4.0 | .053777778000 | .089742366322 | 1.000 | -.27234934123 | .37990489723 |
|  | 4.5 | .089777778000 | .089742366322 | .998 | -.23634934123 | .41590489723 |
|  | 5.5 | -.175111111000 | .089742366322 | .753 | -.50123823023 | .15101600823 |
|  | 6.0 | -.091111111000 | .089742366322 | .997 | -.41723823023 | .23501600823 |
|  | 6.5 | -.108111111000 | .089742366322 | .989 | -.43423823023 | .21801600823 |
|  | 7.0 | -.249111110667 | .089742366322 | .271 | -.57523822990 | .07701600856 |
|  | 7.5 | -.732111111000^*^ | .089742366322 | .000 | -1.05823823023 | -.40598399177 |
|  | 8.0 | -1.176666666333^*^ | .089742366322 | .000 | -1.50279378556 | -.85053954710 |
|  | 8.5 | -1.147444444000^*^ | .089742366322 | .000 | -1.47357156323 | -.82131732477 |
|  | 9.0 | -.475888888667^*^ | .089742366322 | .001 | -.80201600790 | -.14976176944 |
|  | 9.5 | -.610777777333^*^ | .089742366322 | .000 | -.93690489656 | -.28465065810 |
|  | 10.0 | -1.178222222000^*^ | .089742366322 | .000 | -1.50434934123 | -.85209510277 |
| 5.5 | 4.0 | .228888889000 | .089742366322 | .385 | -.09723823023 | .55501600823 |
|  | 4.5 | .264888889000 | .089742366322 | .200 | -.06123823023 | .59101600823 |
|  | 5.0 | .175111111000 | .089742366322 | .753 | -.15101600823 | .50123823023 |
|  | 6.0 | .084000000000 | .089742366322 | .999 | -.24212711923 | .41012711923 |
|  | 6.5 | .067000000000 | .089742366322 | 1.000 | -.25912711923 | .39312711923 |
|  | 7.0 | -.073999999667 | .089742366322 | 1.000 | -.40012711890 | .25212711956 |
|  | 7.5 | -.557000000000^*^ | .089742366322 | .000 | -.88312711923 | -.23087288077 |
|  | 8.0 | -1.001555555333^*^ | .089742366322 | .000 | -1.32768267456 | -.67542843610 |
|  | 8.5 | -.972333333000^*^ | .089742366322 | .000 | -1.29846045223 | -.64620621377 |
|  | 9.0 | -.300777777667 | .089742366322 | .092 | -.62690489690 | .02534934156 |
|  | 9.5 | -.435666666333^*^ | .089742366322 | .003 | -.76179378556 | -.10953954710 |
|  | 10.0 | -1.003111111000^*^ | .089742366322 | .000 | -1.32923823023 | -.67698399177 |
| 6.0 | 4.0 | .144888889000 | .089742366322 | .910 | -.18123823023 | .47101600823 |
|  | 4.5 | .180888889000 | .089742366322 | .715 | -.14523823023 | .50701600823 |
|  | 5.0 | .091111111000 | .089742366322 | .997 | -.23501600823 | .41723823023 |
|  | 5.5 | -.084000000000 | .089742366322 | .999 | -.41012711923 | .24212711923 |
|  | 6.5 | -.017000000000 | .089742366322 | 1.000 | -.34312711923 | .30912711923 |
|  | 7.0 | -.157999999667 | .089742366322 | .851 | -.48412711890 | .16812711956 |
|  | 7.5 | -.641000000000^*^ | .089742366322 | .000 | -.96712711923 | -.31487288077 |
|  | 8.0 | -1.085555555333^*^ | .089742366322 | .000 | -1.41168267456 | -.75942843610 |
|  | 8.5 | -1.056333333000^*^ | .089742366322 | .000 | -1.38246045223 | -.73020621377 |
|  | 9.0 | -.384777777667^*^ | .089742366322 | .011 | -.71090489690 | -.05865065844 |
|  | 9.5 | -.519666666333^*^ | .089742366322 | .000 | -.84579378556 | -.19353954710 |
|  | 10.0 | -1.087111111000^*^ | .089742366322 | .000 | -1.41323823023 | -.76098399177 |
| 6.5 | 4.0 | .161888889000 | .089742366322 | .831 | -.16423823023 | .48801600823 |
|  | 4.5 | .197888889000 | .089742366322 | .596 | -.12823823023 | .52401600823 |
|  | 5.0 | .108111111000 | .089742366322 | .989 | -.21801600823 | .43423823023 |
|  | 5.5 | -.067000000000 | .089742366322 | 1.000 | -.39312711923 | .25912711923 |
|  | 6.0 | .017000000000 | .089742366322 | 1.000 | -.30912711923 | .34312711923 |
|  | 7.0 | -.140999999667 | .089742366322 | .924 | -.46712711890 | .18512711956 |
|  | 7.5 | -.624000000000^*^ | .089742366322 | .000 | -.95012711923 | -.29787288077 |
|  | 8.0 | -1.068555555333^*^ | .089742366322 | .000 | -1.39468267456 | -.74242843610 |
|  | 8.5 | -1.039333333000^*^ | .089742366322 | .000 | -1.36546045223 | -.71320621377 |
|  | 9.0 | -.367777777667^*^ | .089742366322 | .017 | -.69390489690 | -.04165065844 |
|  | 9.5 | -.502666666333^*^ | .089742366322 | .000 | -.82879378556 | -.17653954710 |
|  | 10.0 | -1.070111111000^*^ | .089742366322 | .000 | -1.39623823023 | -.74398399177 |
| 7.0 | 4.0 | .302888888667 | .089742366322 | .087 | -.02323823056 | .62901600790 |
|  | 4.5 | .338888888667^*^ | .089742366322 | .036 | .01276176944 | .66501600790 |
|  | 5.0 | .249111110667 | .089742366322 | .271 | -.07701600856 | .57523822990 |
|  | 5.5 | .073999999667 | .089742366322 | 1.000 | -.25212711956 | .40012711890 |
|  | 6.0 | .157999999667 | .089742366322 | .851 | -.16812711956 | .48412711890 |
|  | 6.5 | .140999999667 | .089742366322 | .924 | -.18512711956 | .46712711890 |
|  | 7.5 | -.483000000333^*^ | .089742366322 | .001 | -.80912711956 | -.15687288110 |
|  | 8.0 | -.927555555667^*^ | .089742366322 | .000 | -1.25368267490 | -.60142843644 |
|  | 8.5 | -.898333333333^*^ | .089742366322 | .000 | -1.22446045256 | -.57220621410 |
|  | 9.0 | -.226777778000 | .089742366322 | .398 | -.55290489723 | .09934934123 |
|  | 9.5 | -.361666666667^*^ | .089742366322 | .020 | -.68779378590 | -.03553954744 |
|  | 10.0 | -.929111111333^*^ | .089742366322 | .000 | -1.25523823056 | -.60298399210 |
| 7.5 | 4.0 | .785888889000^*^ | .089742366322 | .000 | .45976176977 | 1.11201600823 |
|  | 4.5 | .821888889000^*^ | .089742366322 | .000 | .49576176977 | 1.14801600823 |
|  | 5.0 | .732111111000^*^ | .089742366322 | .000 | .40598399177 | 1.05823823023 |
|  | 5.5 | .557000000000^*^ | .089742366322 | .000 | .23087288077 | .88312711923 |
|  | 6.0 | .641000000000^*^ | .089742366322 | .000 | .31487288077 | .96712711923 |
|  | 6.5 | .624000000000^*^ | .089742366322 | .000 | .29787288077 | .95012711923 |
|  | 7.0 | .483000000333^*^ | .089742366322 | .001 | .15687288110 | .80912711956 |
|  | 8.0 | -.444555555333^*^ | .089742366322 | .002 | -.77068267456 | -.11842843610 |
|  | 8.5 | -.415333333000^*^ | .089742366322 | .005 | -.74146045223 | -.08920621377 |
|  | 9.0 | .256222222333 | .089742366322 | .237 | -.06990489690 | .58234934156 |
|  | 9.5 | .121333333667 | .089742366322 | .973 | -.20479378556 | .44746045290 |
|  | 10.0 | -.446111111000^*^ | .089742366322 | .002 | -.77223823023 | -.11998399177 |
| 8.0 | 4.0 | 1.230444444333^*^ | .089742366322 | .000 | .90431732510 | 1.55657156356 |
|  | 4.5 | 1.266444444333^*^ | .089742366322 | .000 | .94031732510 | 1.59257156356 |
|  | 5.0 | 1.176666666333^*^ | .089742366322 | .000 | .85053954710 | 1.50279378556 |
|  | 5.5 | 1.001555555333^*^ | .089742366322 | .000 | .67542843610 | 1.32768267456 |
|  | 6.0 | 1.085555555333^*^ | .089742366322 | .000 | .75942843610 | 1.41168267456 |
|  | 6.5 | 1.068555555333^*^ | .089742366322 | .000 | .74242843610 | 1.39468267456 |
|  | 7.0 | .927555555667^*^ | .089742366322 | .000 | .60142843644 | 1.25368267490 |
|  | 7.5 | .444555555333^*^ | .089742366322 | .002 | .11842843610 | .77068267456 |
|  | 8.5 | .029222222333 | .089742366322 | 1.000 | -.29690489690 | .35534934156 |
|  | 9.0 | .700777777667^*^ | .089742366322 | .000 | .37465065844 | 1.02690489690 |
|  | 9.5 | .565888889000^*^ | .089742366322 | .000 | .23976176977 | .89201600823 |
|  | 10.0 | -.001555555667 | .089742366322 | 1.000 | -.32768267490 | .32457156356 |
| 8.5 | 4.0 | 1.201222222000^*^ | .089742366322 | .000 | .87509510277 | 1.52734934123 |
|  | 4.5 | 1.237222222000^*^ | .089742366322 | .000 | .91109510277 | 1.56334934123 |
|  | 5.0 | 1.147444444000^*^ | .089742366322 | .000 | .82131732477 | 1.47357156323 |
|  | 5.5 | .972333333000^*^ | .089742366322 | .000 | .64620621377 | 1.29846045223 |
|  | 6.0 | 1.056333333000^*^ | .089742366322 | .000 | .73020621377 | 1.38246045223 |
|  | 6.5 | 1.039333333000^*^ | .089742366322 | .000 | .71320621377 | 1.36546045223 |
|  | 7.0 | .898333333333^*^ | .089742366322 | .000 | .57220621410 | 1.22446045256 |
|  | 7.5 | .415333333000^*^ | .089742366322 | .005 | .08920621377 | .74146045223 |
|  | 8.0 | -.029222222333 | .089742366322 | 1.000 | -.35534934156 | .29690489690 |
|  | 9.0 | .671555555333^*^ | .089742366322 | .000 | .34542843610 | .99768267456 |
|  | 9.5 | .536666666667^*^ | .089742366322 | .000 | .21053954744 | .86279378590 |
|  | 10.0 | -.030777778000 | .089742366322 | 1.000 | -.35690489723 | .29534934123 |
| 9.0 | 4.0 | .529666666667^*^ | .089742366322 | .000 | .20353954744 | .85579378590 |
|  | 4.5 | .565666666667^*^ | .089742366322 | .000 | .23953954744 | .89179378590 |
|  | 5.0 | .475888888667^*^ | .089742366322 | .001 | .14976176944 | .80201600790 |
|  | 5.5 | .300777777667 | .089742366322 | .092 | -.02534934156 | .62690489690 |
|  | 6.0 | .384777777667^*^ | .089742366322 | .011 | .05865065844 | .71090489690 |
|  | 6.5 | .367777777667^*^ | .089742366322 | .017 | .04165065844 | .69390489690 |
|  | 7.0 | .226777778000 | .089742366322 | .398 | -.09934934123 | .55290489723 |
|  | 7.5 | -.256222222333 | .089742366322 | .237 | -.58234934156 | .06990489690 |
|  | 8.0 | -.700777777667^*^ | .089742366322 | .000 | -1.02690489690 | -.37465065844 |
|  | 8.5 | -.671555555333^*^ | .089742366322 | .000 | -.99768267456 | -.34542843610 |
|  | 9.5 | -.134888888667 | .089742366322 | .943 | -.46101600790 | .19123823056 |
|  | 10.0 | -.702333333333^*^ | .089742366322 | .000 | -1.02846045256 | -.37620621410 |
| 9.5 | 4.0 | .664555555333^*^ | .089742366322 | .000 | .33842843610 | .99068267456 |
|  | 4.5 | .700555555333^*^ | .089742366322 | .000 | .37442843610 | 1.02668267456 |
|  | 5.0 | .610777777333^*^ | .089742366322 | .000 | .28465065810 | .93690489656 |
|  | 5.5 | .435666666333^*^ | .089742366322 | .003 | .10953954710 | .76179378556 |
|  | 6.0 | .519666666333^*^ | .089742366322 | .000 | .19353954710 | .84579378556 |
|  | 6.5 | .502666666333^*^ | .089742366322 | .000 | .17653954710 | .82879378556 |
|  | 7.0 | .361666666667^*^ | .089742366322 | .020 | .03553954744 | .68779378590 |
|  | 7.5 | -.121333333667 | .089742366322 | .973 | -.44746045290 | .20479378556 |
|  | 8.0 | -.565888889000^*^ | .089742366322 | .000 | -.89201600823 | -.23976176977 |
|  | 8.5 | -.536666666667^*^ | .089742366322 | .000 | -.86279378590 | -.21053954744 |
|  | 9.0 | .134888888667 | .089742366322 | .943 | -.19123823056 | .46101600790 |
|  | 10.0 | -.567444444667^*^ | .089742366322 | .000 | -.89357156390 | -.24131732544 |
| 10.0 | 4.0 | 1.232000000000^*^ | .089742366322 | .000 | .90587288077 | 1.55812711923 |
|  | 4.5 | 1.268000000000^*^ | .089742366322 | .000 | .94187288077 | 1.59412711923 |
|  | 5.0 | 1.178222222000^*^ | .089742366322 | .000 | .85209510277 | 1.50434934123 |
|  | 5.5 | 1.003111111000^*^ | .089742366322 | .000 | .67698399177 | 1.32923823023 |
|  | 6.0 | 1.087111111000^*^ | .089742366322 | .000 | .76098399177 | 1.41323823023 |
|  | 6.5 | 1.070111111000^*^ | .089742366322 | .000 | .74398399177 | 1.39623823023 |
|  | 7.0 | .929111111333^*^ | .089742366322 | .000 | .60298399210 | 1.25523823056 |
|  | 7.5 | .446111111000^*^ | .089742366322 | .002 | .11998399177 | .77223823023 |
|  | 8.0 | .001555555667 | .089742366322 | 1.000 | -.32457156356 | .32768267490 |
|  | 8.5 | .030777778000 | .089742366322 | 1.000 | -.29534934123 | .35690489723 |
|  | 9.0 | .702333333333^*^ | .089742366322 | .000 | .37620621410 | 1.02846045256 |
|  | 9.5 | .567444444667^*^ | .089742366322 | .000 | .24131732544 | .89357156390 |
| *. The mean difference is significant at the 0.05 level. | | | | | | |

**Supplementary table 3a: ANOVA of the Effect of temperature on the activity of immobilized BgaC**

| ANOVA effect of temperature on the activity of Immobilized BgaC | | | | |  |  |
| --- | --- | --- | --- | --- | --- | --- |
| *Source of Variation* | *SS* | *Df* | *MS* | *F* | *P-value* | *F crit* |
| Between Groups | 16.80696 | 9 | 1.86744 | 7.753349 | 7.48E-05 | 2.392814 |
| Within Groups | 4.817119 | 20 | 0.240856 |  |  |  |
|  |  |  |  |  |  |  |
| Total | 21.62408 | 29 |  |  |  |  |

**Supplementary table 3b: Tukey's HSD Results: Effect of Temperature on Activity of Immobilized BgaC**

| **Multiple Comparisons** | | | | | | |
| --- | --- | --- | --- | --- | --- | --- |
| Dependent Variable: 420 nm  Tukey HSD | | | | | | |
| (I) Temperature(oC) | (J) Temperature(oC) | Mean Difference (I-J) | Std. Error | Sig. | 95% Confidence Interval | |
|  |  |  |  |  | Lower Bound | Upper Bound |
| 0 | 10 | -.395000000000 | .400712636748 | .990 | -1.81396664129 | 1.02396664129 |
|  | 20 | -.557222222000 | .400712636748 | .916 | -1.97618886329 | .86174441929 |
|  | 30 | -1.530277778000^*^ | .400712636748 | .028 | -2.94924441929 | -.11131113671 |
|  | 37 | -1.657222222333^*^ | .400712636748 | .014 | -3.07618886363 | -.23825558104 |
|  | 40 | -2.183333333333^*^ | .400712636748 | .001 | -3.60229997463 | -.76436669204 |
|  | 45 | -1.916111111333^*^ | .400712636748 | .004 | -3.33507775263 | -.49714447004 |
|  | 50 | -1.334722222667 | .400712636748 | .076 | -2.75368886396 | .08424441863 |
|  | 55 | -.291944444667 | .400712636748 | .999 | -1.71091108596 | 1.12702219663 |
|  | 60 | -.309166666667 | .400712636748 | .998 | -1.72813330796 | 1.10979997463 |
| 10 | 0 | .395000000000 | .400712636748 | .990 | -1.02396664129 | 1.81396664129 |
|  | 20 | -.162222222000 | .400712636748 | 1.000 | -1.58118886329 | 1.25674441929 |
|  | 30 | -1.135277778000 | .400712636748 | .190 | -2.55424441929 | .28368886329 |
|  | 37 | -1.262222222333 | .400712636748 | .108 | -2.68118886363 | .15674441896 |
|  | 40 | -1.788333333333^*^ | .400712636748 | .007 | -3.20729997463 | -.36936669204 |
|  | 45 | -1.521111111333^*^ | .400712636748 | .030 | -2.94007775263 | -.10214447004 |
|  | 50 | -.939722222667 | .400712636748 | .404 | -2.35868886396 | .47924441863 |
|  | 55 | .103055555333 | .400712636748 | 1.000 | -1.31591108596 | 1.52202219663 |
|  | 60 | .085833333333 | .400712636748 | 1.000 | -1.33313330796 | 1.50479997463 |
| 20 | 0 | .557222222000 | .400712636748 | .916 | -.86174441929 | 1.97618886329 |
|  | 10 | .162222222000 | .400712636748 | 1.000 | -1.25674441929 | 1.58118886329 |
|  | 30 | -.973055556000 | .400712636748 | .360 | -2.39202219729 | .44591108529 |
|  | 37 | -1.100000000333 | .400712636748 | .221 | -2.51896664163 | .31896664096 |
|  | 40 | -1.626111111333^*^ | .400712636748 | .017 | -3.04507775263 | -.20714447004 |
|  | 45 | -1.358888889333 | .400712636748 | .068 | -2.77785553063 | .06007775196 |
|  | 50 | -.777500000667 | .400712636748 | .645 | -2.19646664196 | .64146664063 |
|  | 55 | .265277777333 | .400712636748 | .999 | -1.15368886396 | 1.68424441863 |
|  | 60 | .248055555333 | .400712636748 | 1.000 | -1.17091108596 | 1.66702219663 |
| 30 | 0 | 1.530277778000^*^ | .400712636748 | .028 | .11131113671 | 2.94924441929 |
|  | 10 | 1.135277778000 | .400712636748 | .190 | -.28368886329 | 2.55424441929 |
|  | 20 | .973055556000 | .400712636748 | .360 | -.44591108529 | 2.39202219729 |
|  | 37 | -.126944444333 | .400712636748 | 1.000 | -1.54591108563 | 1.29202219696 |
|  | 40 | -.653055555333 | .400712636748 | .819 | -2.07202219663 | .76591108596 |
|  | 45 | -.385833333333 | .400712636748 | .991 | -1.80479997463 | 1.03313330796 |
|  | 50 | .195555555333 | .400712636748 | 1.000 | -1.22341108596 | 1.61452219663 |
|  | 55 | 1.238333333333 | .400712636748 | .120 | -.18063330796 | 2.65729997463 |
|  | 60 | 1.221111111333 | .400712636748 | .130 | -.19785552996 | 2.64007775263 |
| 37 | 0 | 1.657222222333^*^ | .400712636748 | .014 | .23825558104 | 3.07618886363 |
|  | 10 | 1.262222222333 | .400712636748 | .108 | -.15674441896 | 2.68118886363 |
|  | 20 | 1.100000000333 | .400712636748 | .221 | -.31896664096 | 2.51896664163 |
|  | 30 | .126944444333 | .400712636748 | 1.000 | -1.29202219696 | 1.54591108563 |
|  | 40 | -.526111111000 | .400712636748 | .939 | -1.94507775229 | .89285553029 |
|  | 45 | -.258888889000 | .400712636748 | 1.000 | -1.67785553029 | 1.16007775229 |
|  | 50 | .322499999667 | .400712636748 | .998 | -1.09646664163 | 1.74146664096 |
|  | 55 | 1.365277777667 | .400712636748 | .065 | -.05368886363 | 2.78424441896 |
|  | 60 | 1.348055555667 | .400712636748 | .071 | -.07091108563 | 2.76702219696 |
| 40 | 0 | 2.183333333333^*^ | .400712636748 | .001 | .76436669204 | 3.60229997463 |
|  | 10 | 1.788333333333^*^ | .400712636748 | .007 | .36936669204 | 3.20729997463 |
|  | 20 | 1.626111111333^*^ | .400712636748 | .017 | .20714447004 | 3.04507775263 |
|  | 30 | .653055555333 | .400712636748 | .819 | -.76591108596 | 2.07202219663 |
|  | 37 | .526111111000 | .400712636748 | .939 | -.89285553029 | 1.94507775229 |
|  | 45 | .267222222000 | .400712636748 | .999 | -1.15174441929 | 1.68618886329 |
|  | 50 | .848611110667 | .400712636748 | .536 | -.57035553063 | 2.26757775196 |
|  | 55 | 1.891388888667^*^ | .400712636748 | .004 | .47242224737 | 3.31035552996 |
|  | 60 | 1.874166666667^*^ | .400712636748 | .004 | .45520002537 | 3.29313330796 |
| 45 | 0 | 1.916111111333^*^ | .400712636748 | .004 | .49714447004 | 3.33507775263 |
|  | 10 | 1.521111111333^*^ | .400712636748 | .030 | .10214447004 | 2.94007775263 |
|  | 20 | 1.358888889333 | .400712636748 | .068 | -.06007775196 | 2.77785553063 |
|  | 30 | .385833333333 | .400712636748 | .991 | -1.03313330796 | 1.80479997463 |
|  | 37 | .258888889000 | .400712636748 | 1.000 | -1.16007775229 | 1.67785553029 |
|  | 40 | -.267222222000 | .400712636748 | .999 | -1.68618886329 | 1.15174441929 |
|  | 50 | .581388888667 | .400712636748 | .896 | -.83757775263 | 2.00035552996 |
|  | 55 | 1.624166666667^*^ | .400712636748 | .017 | .20520002537 | 3.04313330796 |
|  | 60 | 1.606944444667^*^ | .400712636748 | .019 | .18797780337 | 3.02591108596 |
| 50 | 0 | 1.334722222667 | .400712636748 | .076 | -.08424441863 | 2.75368886396 |
|  | 10 | .939722222667 | .400712636748 | .404 | -.47924441863 | 2.35868886396 |
|  | 20 | .777500000667 | .400712636748 | .645 | -.64146664063 | 2.19646664196 |
|  | 30 | -.195555555333 | .400712636748 | 1.000 | -1.61452219663 | 1.22341108596 |
|  | 37 | -.322499999667 | .400712636748 | .998 | -1.74146664096 | 1.09646664163 |
|  | 40 | -.848611110667 | .400712636748 | .536 | -2.26757775196 | .57035553063 |
|  | 45 | -.581388888667 | .400712636748 | .896 | -2.00035552996 | .83757775263 |
|  | 55 | 1.042777778000 | .400712636748 | .278 | -.37618886329 | 2.46174441929 |
|  | 60 | 1.025555556000 | .400712636748 | .297 | -.39341108529 | 2.44452219729 |
| 55 | 0 | .291944444667 | .400712636748 | .999 | -1.12702219663 | 1.71091108596 |
|  | 10 | -.103055555333 | .400712636748 | 1.000 | -1.52202219663 | 1.31591108596 |
|  | 20 | -.265277777333 | .400712636748 | .999 | -1.68424441863 | 1.15368886396 |
|  | 30 | -1.238333333333 | .400712636748 | .120 | -2.65729997463 | .18063330796 |
|  | 37 | -1.365277777667 | .400712636748 | .065 | -2.78424441896 | .05368886363 |
|  | 40 | -1.891388888667^*^ | .400712636748 | .004 | -3.31035552996 | -.47242224737 |
|  | 45 | -1.624166666667^*^ | .400712636748 | .017 | -3.04313330796 | -.20520002537 |
|  | 50 | -1.042777778000 | .400712636748 | .278 | -2.46174441929 | .37618886329 |
|  | 60 | -.017222222000 | .400712636748 | 1.000 | -1.43618886329 | 1.40174441929 |
| 60 | 0 | .309166666667 | .400712636748 | .998 | -1.10979997463 | 1.72813330796 |
|  | 10 | -.085833333333 | .400712636748 | 1.000 | -1.50479997463 | 1.33313330796 |
|  | 20 | -.248055555333 | .400712636748 | 1.000 | -1.66702219663 | 1.17091108596 |
|  | 30 | -1.221111111333 | .400712636748 | .130 | -2.64007775263 | .19785552996 |
|  | 37 | -1.348055555667 | .400712636748 | .071 | -2.76702219696 | .07091108563 |
|  | 40 | -1.874166666667^*^ | .400712636748 | .004 | -3.29313330796 | -.45520002537 |
|  | 45 | -1.606944444667^*^ | .400712636748 | .019 | -3.02591108596 | -.18797780337 |
|  | 50 | -1.025555556000 | .400712636748 | .297 | -2.44452219729 | .39341108529 |
|  | 55 | .017222222000 | .400712636748 | 1.000 | -1.40174441929 | 1.43618886329 |
| *. The mean difference is significant at the 0.05 level. | | | | | | |

**Supplementary table 3c: ANOVA of the Effect of temperature on the activity of Free BgaC**

| ANOVA -Effect of Temperature on the activity of Free BgaC | | | | | |  |
| --- | --- | --- | --- | --- | --- | --- |
| *Source of Variation* | *SS* | *df* | *MS* | *F* | *P-value* | *F crit* |
| Between Groups | 1.255327 | 8 | 0.156916 | 14.49998 | 2.13E-06 | 2.510158 |
| Within Groups | 0.194792 | 18 | 0.010822 |  |  |  |
|  |  |  |  |  |  |  |
| Total | 1.450119 | 26 |  |  |  |  |

**Supplementary table 3d: Tukey's HSD Results: Effect of Temperature on Activity of Free BgaC**

| **Multiple Comparisons** | | | | | | |
| --- | --- | --- | --- | --- | --- | --- |
| Dependent Variable: Absorbance  Tukey HSD | | | | | | |
| (I) Temperature (oC) | (J) Temperature (oC) | Mean Difference (I-J) | Std. Error | Sig. | 95% Confidence Interval | |
|  |  |  |  |  | Lower Bound | Upper Bound |
| 0 | 10 | -.063666666333 | .084938402521 | .997 | -.36127911278 | .23394578011 |
|  | 20 | -.377888888667^*^ | .084938402521 | .007 | -.67550133511 | -.08027644222 |
|  | 30 | -.379111111000^*^ | .084938402521 | .007 | -.67672355745 | -.08149866455 |
|  | 37 | -.430777777667^*^ | .084938402521 | .002 | -.72839022411 | -.13316533122 |
|  | 40 | -.207777777333 | .084938402521 | .318 | -.50539022378 | .08983466911 |
|  | 50 | -.022222222000 | .084938402521 | 1.000 | -.31983466845 | .27539022445 |
|  | 55 | .092222222667 | .084938402521 | .969 | -.20539022378 | .38983466911 |
|  | 60 | .210777778000 | .084938402521 | .302 | -.08683466845 | .50839022445 |
| 10 | 0 | .063666666333 | .084938402521 | .997 | -.23394578011 | .36127911278 |
|  | 20 | -.314222222333^*^ | .084938402521 | .034 | -.61183466878 | -.01660977589 |
|  | 30 | -.315444444667^*^ | .084938402521 | .033 | -.61305689111 | -.01783199822 |
|  | 37 | -.367111111333^*^ | .084938402521 | .010 | -.66472355778 | -.06949866489 |
|  | 40 | -.144111111000 | .084938402521 | .741 | -.44172355745 | .15350133545 |
|  | 50 | .041444444333 | .084938402521 | 1.000 | -.25616800211 | .33905689078 |
|  | 55 | .155888889000 | .084938402521 | .661 | -.14172355745 | .45350133545 |
|  | 60 | .274444444333 | .084938402521 | .084 | -.02316800211 | .57205689078 |
| 20 | 0 | .377888888667^*^ | .084938402521 | .007 | .08027644222 | .67550133511 |
|  | 10 | .314222222333^*^ | .084938402521 | .034 | .01660977589 | .61183466878 |
|  | 30 | -.001222222333 | .084938402521 | 1.000 | -.29883466878 | .29639022411 |
|  | 37 | -.052888889000 | .084938402521 | .999 | -.35050133545 | .24472355745 |
|  | 40 | .170111111333 | .084938402521 | .560 | -.12750133511 | .46772355778 |
|  | 50 | .355666666667^*^ | .084938402521 | .013 | .05805422022 | .65327911311 |
|  | 55 | .470111111333^*^ | .084938402521 | .001 | .17249866489 | .76772355778 |
|  | 60 | .588666666667^*^ | .084938402521 | .000 | .29105422022 | .88627911311 |
| 30 | 0 | .379111111000^*^ | .084938402521 | .007 | .08149866455 | .67672355745 |
|  | 10 | .315444444667^*^ | .084938402521 | .033 | .01783199822 | .61305689111 |
|  | 20 | .001222222333 | .084938402521 | 1.000 | -.29639022411 | .29883466878 |
|  | 37 | -.051666666667 | .084938402521 | .999 | -.34927911311 | .24594577978 |
|  | 40 | .171333333667 | .084938402521 | .551 | -.12627911278 | .46894578011 |
|  | 50 | .356888889000^*^ | .084938402521 | .012 | .05927644255 | .65450133545 |
|  | 55 | .471333333667^*^ | .084938402521 | .001 | .17372088722 | .76894578011 |
|  | 60 | .589888889000^*^ | .084938402521 | .000 | .29227644255 | .88750133545 |
| 37 | 0 | .430777777667^*^ | .084938402521 | .002 | .13316533122 | .72839022411 |
|  | 10 | .367111111333^*^ | .084938402521 | .010 | .06949866489 | .66472355778 |
|  | 20 | .052888889000 | .084938402521 | .999 | -.24472355745 | .35050133545 |
|  | 30 | .051666666667 | .084938402521 | .999 | -.24594577978 | .34927911311 |
|  | 40 | .223000000333 | .084938402521 | .242 | -.07461244611 | .52061244678 |
|  | 50 | .408555555667^*^ | .084938402521 | .003 | .11094310922 | .70616800211 |
|  | 55 | .523000000333^*^ | .084938402521 | .000 | .22538755389 | .82061244678 |
|  | 60 | .641555555667^*^ | .084938402521 | .000 | .34394310922 | .93916800211 |
| 40 | 0 | .207777777333 | .084938402521 | .318 | -.08983466911 | .50539022378 |
|  | 10 | .144111111000 | .084938402521 | .741 | -.15350133545 | .44172355745 |
|  | 20 | -.170111111333 | .084938402521 | .560 | -.46772355778 | .12750133511 |
|  | 30 | -.171333333667 | .084938402521 | .551 | -.46894578011 | .12627911278 |
|  | 37 | -.223000000333 | .084938402521 | .242 | -.52061244678 | .07461244611 |
|  | 50 | .185555555333 | .084938402521 | .454 | -.11205689111 | .48316800178 |
|  | 55 | .300000000000^*^ | .084938402521 | .047 | .00238755355 | .59761244645 |
|  | 60 | .418555555333^*^ | .084938402521 | .003 | .12094310889 | .71616800178 |
| 50 | 0 | .022222222000 | .084938402521 | 1.000 | -.27539022445 | .31983466845 |
|  | 10 | -.041444444333 | .084938402521 | 1.000 | -.33905689078 | .25616800211 |
|  | 20 | -.355666666667^*^ | .084938402521 | .013 | -.65327911311 | -.05805422022 |
|  | 30 | -.356888889000^*^ | .084938402521 | .012 | -.65450133545 | -.05927644255 |
|  | 37 | -.408555555667^*^ | .084938402521 | .003 | -.70616800211 | -.11094310922 |
|  | 40 | -.185555555333 | .084938402521 | .454 | -.48316800178 | .11205689111 |
|  | 55 | .114444444667 | .084938402521 | .903 | -.18316800178 | .41205689111 |
|  | 60 | .233000000000 | .084938402521 | .200 | -.06461244645 | .53061244645 |
| 55 | 0 | -.092222222667 | .084938402521 | .969 | -.38983466911 | .20539022378 |
|  | 10 | -.155888889000 | .084938402521 | .661 | -.45350133545 | .14172355745 |
|  | 20 | -.470111111333^*^ | .084938402521 | .001 | -.76772355778 | -.17249866489 |
|  | 30 | -.471333333667^*^ | .084938402521 | .001 | -.76894578011 | -.17372088722 |
|  | 37 | -.523000000333^*^ | .084938402521 | .000 | -.82061244678 | -.22538755389 |
|  | 40 | -.300000000000^*^ | .084938402521 | .047 | -.59761244645 | -.00238755355 |
|  | 50 | -.114444444667 | .084938402521 | .903 | -.41205689111 | .18316800178 |
|  | 60 | .118555555333 | .084938402521 | .885 | -.17905689111 | .41616800178 |
| 60 | 0 | -.210777778000 | .084938402521 | .302 | -.50839022445 | .08683466845 |
|  | 10 | -.274444444333 | .084938402521 | .084 | -.57205689078 | .02316800211 |
|  | 20 | -.588666666667^*^ | .084938402521 | .000 | -.88627911311 | -.29105422022 |
|  | 30 | -.589888889000^*^ | .084938402521 | .000 | -.88750133545 | -.29227644255 |
|  | 37 | -.641555555667^*^ | .084938402521 | .000 | -.93916800211 | -.34394310922 |
|  | 40 | -.418555555333^*^ | .084938402521 | .003 | -.71616800178 | -.12094310889 |
|  | 50 | -.233000000000 | .084938402521 | .200 | -.53061244645 | .06461244645 |
|  | 55 | -.118555555333 | .084938402521 | .885 | -.41616800178 | .17905689111 |
| *. The mean difference is significant at the 0.05 level. | | | | | | |

**Supplementary table 4a: ANOVA of the Effect of temperature on the stability of immobilized BgaC**

| ANOVA-Effect of Temperature on stability of immobilized BgaC | | | | | | |
| --- | --- | --- | --- | --- | --- | --- |
| *Source of Variation* | *SS* | *df* | *MS* | *F* | *P-value* | *F crit* |
| Between Groups | 32.87811 | 9 | 3.653123 | 204.0602 | 1.11E-17 | 2.392814 |
| Within Groups | 0.358044 | 20 | 0.017902 |  |  |  |
|  |  |  |  |  |  |  |
| Total | 33.23615 | 29 |  |  |  |  |

**Supplementary table 4b:Tukey's HSD Results: Effect of Temperature on Stability of Immobilized BgaC**

| **Multiple Comparisons** | | | | | | |
| --- | --- | --- | --- | --- | --- | --- |
| Dependent Variable: 420nm  Tukey HSD | | | | | | |
| (I) oC | (J) oC | Mean Difference (I-J) | Std. Error | Sig. | 95% Confidence Interval | |
|  |  |  |  |  | Lower Bound | Upper Bound |
| 0 | 10 | .521666666333^*^ | .109246471363 | .004 | .13481313508 | .90852019758 |
|  | 20 | .239444444000 | .109246471363 | .492 | -.14740908725 | .62629797525 |
|  | 30 | .188333333333 | .109246471363 | .771 | -.19852019792 | .57518686458 |
|  | 37 | -.101111111333 | .109246471363 | .993 | -.48796464258 | .28574241992 |
|  | 40 | .541388888667^*^ | .109246471363 | .002 | .15453535742 | .92824241992 |
|  | 45 | 2.194722222333^*^ | .109246471363 | .000 | 1.80786869108 | 2.58157575358 |
|  | 50 | 2.369166666333^*^ | .109246471363 | .000 | 1.98231313508 | 2.75602019758 |
|  | 55 | 2.382972222000^*^ | .109246471363 | .000 | 1.99611869075 | 2.76982575325 |
|  | 60 | 2.381111111000^*^ | .109246471363 | .000 | 1.99425757975 | 2.76796464225 |
| 10 | 0 | -.521666666333^*^ | .109246471363 | .004 | -.90852019758 | -.13481313508 |
|  | 20 | -.282222222333 | .109246471363 | .286 | -.66907575358 | .10463130892 |
|  | 30 | -.333333333000 | .109246471363 | .129 | -.72018686425 | .05352019825 |
|  | 37 | -.622777777667^*^ | .109246471363 | .000 | -1.00963130892 | -.23592424642 |
|  | 40 | .019722222333 | .109246471363 | 1.000 | -.36713130892 | .40657575358 |
|  | 45 | 1.673055556000^*^ | .109246471363 | .000 | 1.28620202475 | 2.05990908725 |
|  | 50 | 1.847500000000^*^ | .109246471363 | .000 | 1.46064646875 | 2.23435353125 |
|  | 55 | 1.861305555667^*^ | .109246471363 | .000 | 1.47445202442 | 2.24815908692 |
|  | 60 | 1.859444444667^*^ | .109246471363 | .000 | 1.47259091342 | 2.24629797592 |
| 20 | 0 | -.239444444000 | .109246471363 | .492 | -.62629797525 | .14740908725 |
|  | 10 | .282222222333 | .109246471363 | .286 | -.10463130892 | .66907575358 |
|  | 30 | -.051111110667 | .109246471363 | 1.000 | -.43796464192 | .33574242058 |
|  | 37 | -.340555555333 | .109246471363 | .114 | -.72740908658 | .04629797592 |
|  | 40 | .301944444667 | .109246471363 | .214 | -.08490908658 | .68879797592 |
|  | 45 | 1.955277778333^*^ | .109246471363 | .000 | 1.56842424708 | 2.34213130958 |
|  | 50 | 2.129722222333^*^ | .109246471363 | .000 | 1.74286869108 | 2.51657575358 |
|  | 55 | 2.143527778000^*^ | .109246471363 | .000 | 1.75667424675 | 2.53038130925 |
|  | 60 | 2.141666667000^*^ | .109246471363 | .000 | 1.75481313575 | 2.52852019825 |
| 30 | 0 | -.188333333333 | .109246471363 | .771 | -.57518686458 | .19852019792 |
|  | 10 | .333333333000 | .109246471363 | .129 | -.05352019825 | .72018686425 |
|  | 20 | .051111110667 | .109246471363 | 1.000 | -.33574242058 | .43796464192 |
|  | 37 | -.289444444667 | .109246471363 | .258 | -.67629797592 | .09740908658 |
|  | 40 | .353055555333 | .109246471363 | .092 | -.03379797592 | .73990908658 |
|  | 45 | 2.006388889000^*^ | .109246471363 | .000 | 1.61953535775 | 2.39324242025 |
|  | 50 | 2.180833333000^*^ | .109246471363 | .000 | 1.79397980175 | 2.56768686425 |
|  | 55 | 2.194638888667^*^ | .109246471363 | .000 | 1.80778535742 | 2.58149241992 |
|  | 60 | 2.192777777667^*^ | .109246471363 | .000 | 1.80592424642 | 2.57963130892 |
| 37 | 0 | .101111111333 | .109246471363 | .993 | -.28574241992 | .48796464258 |
|  | 10 | .622777777667^*^ | .109246471363 | .000 | .23592424642 | 1.00963130892 |
|  | 20 | .340555555333 | .109246471363 | .114 | -.04629797592 | .72740908658 |
|  | 30 | .289444444667 | .109246471363 | .258 | -.09740908658 | .67629797592 |
|  | 40 | .642500000000^*^ | .109246471363 | .000 | .25564646875 | 1.02935353125 |
|  | 45 | 2.295833333667^*^ | .109246471363 | .000 | 1.90897980242 | 2.68268686492 |
|  | 50 | 2.470277777667^*^ | .109246471363 | .000 | 2.08342424642 | 2.85713130892 |
|  | 55 | 2.484083333333^*^ | .109246471363 | .000 | 2.09722980208 | 2.87093686458 |
|  | 60 | 2.482222222333^*^ | .109246471363 | .000 | 2.09536869108 | 2.86907575358 |
| 40 | 0 | -.541388888667^*^ | .109246471363 | .002 | -.92824241992 | -.15453535742 |
|  | 10 | -.019722222333 | .109246471363 | 1.000 | -.40657575358 | .36713130892 |
|  | 20 | -.301944444667 | .109246471363 | .214 | -.68879797592 | .08490908658 |
|  | 30 | -.353055555333 | .109246471363 | .092 | -.73990908658 | .03379797592 |
|  | 37 | -.642500000000^*^ | .109246471363 | .000 | -1.02935353125 | -.25564646875 |
|  | 45 | 1.653333333667^*^ | .109246471363 | .000 | 1.26647980242 | 2.04018686492 |
|  | 50 | 1.827777777667^*^ | .109246471363 | .000 | 1.44092424642 | 2.21463130892 |
|  | 55 | 1.841583333333^*^ | .109246471363 | .000 | 1.45472980208 | 2.22843686458 |
|  | 60 | 1.839722222333^*^ | .109246471363 | .000 | 1.45286869108 | 2.22657575358 |
| 45 | 0 | -2.194722222333^*^ | .109246471363 | .000 | -2.58157575358 | -1.80786869108 |
|  | 10 | -1.673055556000^*^ | .109246471363 | .000 | -2.05990908725 | -1.28620202475 |
|  | 20 | -1.955277778333^*^ | .109246471363 | .000 | -2.34213130958 | -1.56842424708 |
|  | 30 | -2.006388889000^*^ | .109246471363 | .000 | -2.39324242025 | -1.61953535775 |
|  | 37 | -2.295833333667^*^ | .109246471363 | .000 | -2.68268686492 | -1.90897980242 |
|  | 40 | -1.653333333667^*^ | .109246471363 | .000 | -2.04018686492 | -1.26647980242 |
|  | 50 | .174444444000 | .109246471363 | .835 | -.21240908725 | .56129797525 |
|  | 55 | .188249999667 | .109246471363 | .771 | -.19860353158 | .57510353092 |
|  | 60 | .186388888667 | .109246471363 | .780 | -.20046464258 | .57324241992 |
| 50 | 0 | -2.369166666333^*^ | .109246471363 | .000 | -2.75602019758 | -1.98231313508 |
|  | 10 | -1.847500000000^*^ | .109246471363 | .000 | -2.23435353125 | -1.46064646875 |
|  | 20 | -2.129722222333^*^ | .109246471363 | .000 | -2.51657575358 | -1.74286869108 |
|  | 30 | -2.180833333000^*^ | .109246471363 | .000 | -2.56768686425 | -1.79397980175 |
|  | 37 | -2.470277777667^*^ | .109246471363 | .000 | -2.85713130892 | -2.08342424642 |
|  | 40 | -1.827777777667^*^ | .109246471363 | .000 | -2.21463130892 | -1.44092424642 |
|  | 45 | -.174444444000 | .109246471363 | .835 | -.56129797525 | .21240908725 |
|  | 55 | .013805555667 | .109246471363 | 1.000 | -.37304797558 | .40065908692 |
|  | 60 | .011944444667 | .109246471363 | 1.000 | -.37490908658 | .39879797592 |
| 55 | 0 | -2.382972222000^*^ | .109246471363 | .000 | -2.76982575325 | -1.99611869075 |
|  | 10 | -1.861305555667^*^ | .109246471363 | .000 | -2.24815908692 | -1.47445202442 |
|  | 20 | -2.143527778000^*^ | .109246471363 | .000 | -2.53038130925 | -1.75667424675 |
|  | 30 | -2.194638888667^*^ | .109246471363 | .000 | -2.58149241992 | -1.80778535742 |
|  | 37 | -2.484083333333^*^ | .109246471363 | .000 | -2.87093686458 | -2.09722980208 |
|  | 40 | -1.841583333333^*^ | .109246471363 | .000 | -2.22843686458 | -1.45472980208 |
|  | 45 | -.188249999667 | .109246471363 | .771 | -.57510353092 | .19860353158 |
|  | 50 | -.013805555667 | .109246471363 | 1.000 | -.40065908692 | .37304797558 |
|  | 60 | -.001861111000 | .109246471363 | 1.000 | -.38871464225 | .38499242025 |
| 60 | 0 | -2.381111111000^*^ | .109246471363 | .000 | -2.76796464225 | -1.99425757975 |
|  | 10 | -1.859444444667^*^ | .109246471363 | .000 | -2.24629797592 | -1.47259091342 |
|  | 20 | -2.141666667000^*^ | .109246471363 | .000 | -2.52852019825 | -1.75481313575 |
|  | 30 | -2.192777777667^*^ | .109246471363 | .000 | -2.57963130892 | -1.80592424642 |
|  | 37 | -2.482222222333^*^ | .109246471363 | .000 | -2.86907575358 | -2.09536869108 |
|  | 40 | -1.839722222333^*^ | .109246471363 | .000 | -2.22657575358 | -1.45286869108 |
|  | 45 | -.186388888667 | .109246471363 | .780 | -.57324241992 | .20046464258 |
|  | 50 | -.011944444667 | .109246471363 | 1.000 | -.39879797592 | .37490908658 |
|  | 55 | .001861111000 | .109246471363 | 1.000 | -.38499242025 | .38871464225 |
| *. The mean difference is significant at the 0.05 level. | | | | | | |

**Supplementary table 4c: ANOVA of the Effect of temperature on the stability of free BgaC**

| ANOVA- Effect of Temperature on stability of Free BgaC | | | | | | |
| --- | --- | --- | --- | --- | --- | --- |
| *Source of Variation* | *SS* | *df* | *MS* | *F* | *P-value* | *F crit* |
| Between Groups | 6.432596 | 9 | 0.714733 | 110.0994 | 4.72E-15 | 2.392814 |
| Within Groups | 0.129834 | 20 | 0.006492 |  |  |  |
|  |  |  |  |  |  |  |
| Total | 6.56243 | 29 |  |  |  |  |

**Supplementary table 4d: Tukey's HSD Results: Effect of Temperature on Stability of Free BgaC**

| **Multiple Comparisons** | | | | | | |
| --- | --- | --- | --- | --- | --- | --- |
| Dependent Variable: 420nm  Tukey HSD | | | | | | |
| (I) oc | (J) oc | Mean Difference (I-J) | Std. Error | Sig. | 95% Confidence Interval | |
|  |  |  |  |  | Lower Bound | Upper Bound |
| 0 | 10 | .255444444333^*^ | .065786035557 | .025 | .02248900106 | .48839988760 |
|  | 20 | .116555555667 | .065786035557 | .744 | -.11639988760 | .34951099894 |
|  | 30 | -.016333333333 | .065786035557 | 1.000 | -.24928877660 | .21662210994 |
|  | 37 | -.206222222333 | .065786035557 | .111 | -.43917766560 | .02673322094 |
|  | 40 | .407888889000^*^ | .065786035557 | .000 | .17493344573 | .64084433227 |
|  | 45 | .977888889000^*^ | .065786035557 | .000 | .74493344573 | 1.21084433227 |
|  | 50 | .990555555667^*^ | .065786035557 | .000 | .75760011240 | 1.22351099894 |
|  | 55 | .990777777667^*^ | .065786035557 | .000 | .75782233440 | 1.22373322094 |
|  | 60 | .978666666667^*^ | .065786035557 | .000 | .74571122340 | 1.21162210994 |
| 10 | 0 | -.255444444333^*^ | .065786035557 | .025 | -.48839988760 | -.02248900106 |
|  | 20 | -.138888888667 | .065786035557 | .540 | -.37184433194 | .09406655460 |
|  | 30 | -.271777777667^*^ | .065786035557 | .015 | -.50473322094 | -.03882233440 |
|  | 37 | -.461666666667^*^ | .065786035557 | .000 | -.69462210994 | -.22871122340 |
|  | 40 | .152444444667 | .065786035557 | .420 | -.08051099860 | .38539988794 |
|  | 45 | .722444444667^*^ | .065786035557 | .000 | .48948900140 | .95539988794 |
|  | 50 | .735111111333^*^ | .065786035557 | .000 | .50215566806 | .96806655460 |
|  | 55 | .735333333333^*^ | .065786035557 | .000 | .50237789006 | .96828877660 |
|  | 60 | .723222222333^*^ | .065786035557 | .000 | .49026677906 | .95617766560 |
| 20 | 0 | -.116555555667 | .065786035557 | .744 | -.34951099894 | .11639988760 |
|  | 10 | .138888888667 | .065786035557 | .540 | -.09406655460 | .37184433194 |
|  | 30 | -.132888889000 | .065786035557 | .596 | -.36584433227 | .10006655427 |
|  | 37 | -.322777778000^*^ | .065786035557 | .003 | -.55573322127 | -.08982233473 |
|  | 40 | .291333333333^*^ | .065786035557 | .008 | .05837789006 | .52428877660 |
|  | 45 | .861333333333^*^ | .065786035557 | .000 | .62837789006 | 1.09428877660 |
|  | 50 | .874000000000^*^ | .065786035557 | .000 | .64104455673 | 1.10695544327 |
|  | 55 | .874222222000^*^ | .065786035557 | .000 | .64126677873 | 1.10717766527 |
|  | 60 | .862111111000^*^ | .065786035557 | .000 | .62915566773 | 1.09506655427 |
| 30 | 0 | .016333333333 | .065786035557 | 1.000 | -.21662210994 | .24928877660 |
|  | 10 | .271777777667^*^ | .065786035557 | .015 | .03882233440 | .50473322094 |
|  | 20 | .132888889000 | .065786035557 | .596 | -.10006655427 | .36584433227 |
|  | 37 | -.189888889000 | .065786035557 | .174 | -.42284433227 | .04306655427 |
|  | 40 | .424222222333^*^ | .065786035557 | .000 | .19126677906 | .65717766560 |
|  | 45 | .994222222333^*^ | .065786035557 | .000 | .76126677906 | 1.22717766560 |
|  | 50 | 1.006888889000^*^ | .065786035557 | .000 | .77393344573 | 1.23984433227 |
|  | 55 | 1.007111111000^*^ | .065786035557 | .000 | .77415566773 | 1.24006655427 |
|  | 60 | .995000000000^*^ | .065786035557 | .000 | .76204455673 | 1.22795544327 |
| 37 | 0 | .206222222333 | .065786035557 | .111 | -.02673322094 | .43917766560 |
|  | 10 | .461666666667^*^ | .065786035557 | .000 | .22871122340 | .69462210994 |
|  | 20 | .322777778000^*^ | .065786035557 | .003 | .08982233473 | .55573322127 |
|  | 30 | .189888889000 | .065786035557 | .174 | -.04306655427 | .42284433227 |
|  | 40 | .614111111333^*^ | .065786035557 | .000 | .38115566806 | .84706655460 |
|  | 45 | 1.184111111333^*^ | .065786035557 | .000 | .95115566806 | 1.41706655460 |
|  | 50 | 1.196777778000^*^ | .065786035557 | .000 | .96382233473 | 1.42973322127 |
|  | 55 | 1.197000000000^*^ | .065786035557 | .000 | .96404455673 | 1.42995544327 |
|  | 60 | 1.184888889000^*^ | .065786035557 | .000 | .95193344573 | 1.41784433227 |
| 40 | 0 | -.407888889000^*^ | .065786035557 | .000 | -.64084433227 | -.17493344573 |
|  | 10 | -.152444444667 | .065786035557 | .420 | -.38539988794 | .08051099860 |
|  | 20 | -.291333333333^*^ | .065786035557 | .008 | -.52428877660 | -.05837789006 |
|  | 30 | -.424222222333^*^ | .065786035557 | .000 | -.65717766560 | -.19126677906 |
|  | 37 | -.614111111333^*^ | .065786035557 | .000 | -.84706655460 | -.38115566806 |
|  | 45 | .570000000000^*^ | .065786035557 | .000 | .33704455673 | .80295544327 |
|  | 50 | .582666666667^*^ | .065786035557 | .000 | .34971122340 | .81562210994 |
|  | 55 | .582888888667^*^ | .065786035557 | .000 | .34993344540 | .81584433194 |
|  | 60 | .570777777667^*^ | .065786035557 | .000 | .33782233440 | .80373322094 |
| 45 | 0 | -.977888889000^*^ | .065786035557 | .000 | -1.21084433227 | -.74493344573 |
|  | 10 | -.722444444667^*^ | .065786035557 | .000 | -.95539988794 | -.48948900140 |
|  | 20 | -.861333333333^*^ | .065786035557 | .000 | -1.09428877660 | -.62837789006 |
|  | 30 | -.994222222333^*^ | .065786035557 | .000 | -1.22717766560 | -.76126677906 |
|  | 37 | -1.184111111333^*^ | .065786035557 | .000 | -1.41706655460 | -.95115566806 |
|  | 40 | -.570000000000^*^ | .065786035557 | .000 | -.80295544327 | -.33704455673 |
|  | 50 | .012666666667 | .065786035557 | 1.000 | -.22028877660 | .24562210994 |
|  | 55 | .012888888667 | .065786035557 | 1.000 | -.22006655460 | .24584433194 |
|  | 60 | .000777777667 | .065786035557 | 1.000 | -.23217766560 | .23373322094 |
| 50 | 0 | -.990555555667^*^ | .065786035557 | .000 | -1.22351099894 | -.75760011240 |
|  | 10 | -.735111111333^*^ | .065786035557 | .000 | -.96806655460 | -.50215566806 |
|  | 20 | -.874000000000^*^ | .065786035557 | .000 | -1.10695544327 | -.64104455673 |
|  | 30 | -1.006888889000^*^ | .065786035557 | .000 | -1.23984433227 | -.77393344573 |
|  | 37 | -1.196777778000^*^ | .065786035557 | .000 | -1.42973322127 | -.96382233473 |
|  | 40 | -.582666666667^*^ | .065786035557 | .000 | -.81562210994 | -.34971122340 |
|  | 45 | -.012666666667 | .065786035557 | 1.000 | -.24562210994 | .22028877660 |
|  | 55 | .000222222000 | .065786035557 | 1.000 | -.23273322127 | .23317766527 |
|  | 60 | -.011888889000 | .065786035557 | 1.000 | -.24484433227 | .22106655427 |
| 55 | 0 | -.990777777667^*^ | .065786035557 | .000 | -1.22373322094 | -.75782233440 |
|  | 10 | -.735333333333^*^ | .065786035557 | .000 | -.96828877660 | -.50237789006 |
|  | 20 | -.874222222000^*^ | .065786035557 | .000 | -1.10717766527 | -.64126677873 |
|  | 30 | -1.007111111000^*^ | .065786035557 | .000 | -1.24006655427 | -.77415566773 |
|  | 37 | -1.197000000000^*^ | .065786035557 | .000 | -1.42995544327 | -.96404455673 |
|  | 40 | -.582888888667^*^ | .065786035557 | .000 | -.81584433194 | -.34993344540 |
|  | 45 | -.012888888667 | .065786035557 | 1.000 | -.24584433194 | .22006655460 |
|  | 50 | -.000222222000 | .065786035557 | 1.000 | -.23317766527 | .23273322127 |
|  | 60 | -.012111111000 | .065786035557 | 1.000 | -.24506655427 | .22084433227 |
| 60 | 0 | -.978666666667^*^ | .065786035557 | .000 | -1.21162210994 | -.74571122340 |
|  | 10 | -.723222222333^*^ | .065786035557 | .000 | -.95617766560 | -.49026677906 |
|  | 20 | -.862111111000^*^ | .065786035557 | .000 | -1.09506655427 | -.62915566773 |
|  | 30 | -.995000000000^*^ | .065786035557 | .000 | -1.22795544327 | -.76204455673 |
|  | 37 | -1.184888889000^*^ | .065786035557 | .000 | -1.41784433227 | -.95193344573 |
|  | 40 | -.570777777667^*^ | .065786035557 | .000 | -.80373322094 | -.33782233440 |
|  | 45 | -.000777777667 | .065786035557 | 1.000 | -.23373322094 | .23217766560 |
|  | 50 | .011888889000 | .065786035557 | 1.000 | -.22106655427 | .24484433227 |
|  | 55 | .012111111000 | .065786035557 | 1.000 | -.22084433227 | .24506655427 |
| *. The mean difference is significant at the 0.05 level. | | | | | | |

**Supplementary Tables 5a. Repeated Measures Analysis of Variance (ANOVA) on the Specific activity of immobilized BgaC.**

| **Within-Subjects Factors** | |
| --- | --- |
| Measure: MEASURE_1 | |
| Round | Dependent Variable |
| 1 | ActR1 |
| 2 | ActR2 |
| 3 | ActR3 |
| 4 | ActR4 |
| 5 | ActR5 |
| 6 | ActR6 |
| 7 | ActR7 |
| 8 | ActR8 |
| 9 | ActR9 |
| 10 | ActR10 |
| 11 | ActR11 |
| 12 | ActR12 |

| **Descriptive Statistics** | | | |
| --- | --- | --- | --- |
|  | Mean | Std. Deviation | N |
| Act-R1 | 51.83 | 9.006 | 3 |
| Act-R2 | 52.64 | 24.580 | 3 |
| Act-R3 | 80.88 | 7.338 | 3 |
| Act-R4 | 43.12 | 25.018 | 3 |
| Act-R5 | 50.73 | 12.910 | 3 |
| Act-R6 | 63.73 | 15.836 | 3 |
| Act-R7 | 50.58 | 13.228 | 3 |
| Act-R8 | 34.19 | 7.246 | 3 |
| Act-R9 | 39.46 | 22.493 | 3 |
| Act-R10 | 43.09 | 26.403 | 3 |
| Act-R11 | 50.55 | 23.470 | 3 |
| Act-R12 | 41.79 | 14.518 | 3 |

| **Multivariate Tests^a^** | | | | | | | | | | |
| --- | --- | --- | --- | --- | --- | --- | --- | --- | --- | --- |
| Effect | | Value | F | Hypothesis df | Error df | Sig. | Partial Eta Squared | Noncent. Parameter | Observed Power^c^ |  |
| Round | Pillai's Trace | .^b^ | . | . | . | . | . | . | . |  |
|  | Wilks' Lambda | .^b^ | . | . | . | . | . | . | . |  |
|  | Hotelling's Trace | .^b^ | . | . | . | . | . | . | . |  |
|  | Roy's Largest Root | .^b^ | . | . | . | . | . | . | . |  |
| a. Design: Intercept  Within Subjects Design: Round | | | | | | | | | | |
| b. Cannot produce multivariate test statistics because of insufficient residual degrees of freedom. | | | | | | | | | | |
| c. Computed using alpha = .05 | | | | | | | | | | |

| **Mauchly's Test of Sphericity^a^** | | | | | | | |
| --- | --- | --- | --- | --- | --- | --- | --- |
| Measure: MEASURE_1 | | | | | | | |
| Within Subjects Effect | Mauchly's W | Approx. Chi-Square | df | Sig. | Epsilon^b^ | | |
|  |  |  |  |  | Greenhouse-Geisser | Huynh-Feldt | Lower-bound |
| Round | .000 | . | 65 | . | .113 | .208 | .091 |
| Tests the null hypothesis that the error covariance matrix of the orthonormalized transformed dependent variables is proportional to an identity matrix. | | | | | | | |
| a. Design: Intercept  Within Subjects Design: Round | | | | | | | |
| b. May be used to adjust the degrees of freedom for the averaged tests of significance. Corrected tests are displayed in the Tests of Within-Subjects Effects table. | | | | | | | |

| **Tests of Within-Subjects Effects** | | | | | | | | | | |
| --- | --- | --- | --- | --- | --- | --- | --- | --- | --- | --- |
| Measure: MEASURE_1 | | | | | | | | | | |
| Source | | Type III Sum of Squares | df | Mean Square | F | Sig. | Partial Eta Squared | Noncent. Parameter | Observed Power^a^ |  |
| Round | Sphericity Assumed | 5029.398 | 11 | 457.218 | 7.134 | .000 | .781 | 78.478 | 1.000 |  |
|  | Greenhouse-Geisser | 5029.398 | 1.243 | 4045.755 | 7.134 | .093 | .781 | 8.869 | .404 |  |
|  | Huynh-Feldt | 5029.398 | 2.285 | 2201.127 | 7.134 | .038 | .781 | 16.301 | .676 |  |
|  | Lower-bound | 5029.398 | 1.000 | 5029.398 | 7.134 | .116 | .781 | 7.134 | .329 |  |
| Error(Round) | Sphericity Assumed | 1409.911 | 22 | 64.087 |  |  |  |  |  |  |
|  | Greenhouse-Geisser | 1409.911 | 2.486 | 567.082 |  |  |  |  |  |  |
|  | Huynh-Feldt | 1409.911 | 4.570 | 308.525 |  |  |  |  |  |  |
|  | Lower-bound | 1409.911 | 2.000 | 704.956 |  |  |  |  |  |  |
| a. Computed using alpha = .05 | | | | | | | | | | |

| **Tests of Within-Subjects Contrasts** | | | | | | | | | |
| --- | --- | --- | --- | --- | --- | --- | --- | --- | --- |
| Measure: MEASURE_1 | | | | | | | | | |
| Source | Round | Type III Sum of Squares | df | Mean Square | F | Sig. | Partial Eta Squared | Noncent. Parameter | Observed Power^a^ |
| Round | Linear | 1182.193 | 1 | 1182.193 | 18.895 | .049 | .904 | 18.895 | .622 |
|  | Quadratic | .053 | 1 | .053 | .005 | .949 | .003 | .005 | .050 |
|  | Cubic | 544.322 | 1 | 544.322 | 102.583 | .010 | .981 | 102.583 | .994 |
|  | Order 4 | 154.180 | 1 | 154.180 | .719 | .486 | .265 | .719 | .083 |
|  | Order 5 | 49.942 | 1 | 49.942 | .987 | .425 | .330 | .987 | .095 |
|  | Order 6 | 316.220 | 1 | 316.220 | 30.173 | .032 | .938 | 30.173 | .782 |
|  | Order 7 | 176.251 | 1 | 176.251 | 1.261 | .378 | .387 | 1.261 | .107 |
|  | Order 8 | 1567.512 | 1 | 1567.512 | 71.043 | .014 | .973 | 71.043 | .970 |
|  | Order 9 | 1033.606 | 1 | 1033.606 | 21.879 | .043 | .916 | 21.879 | .673 |
|  | Order 10 | 2.071 | 1 | 2.071 | .015 | .915 | .007 | .015 | .051 |
|  | Order 11 | 3.049 | 1 | 3.049 | 2.133 | .282 | .516 | 2.133 | .144 |
| Error(Round) | Linear | 125.135 | 2 | 62.567 |  |  |  |  |  |
|  | Quadratic | 20.011 | 2 | 10.006 |  |  |  |  |  |
|  | Cubic | 10.612 | 2 | 5.306 |  |  |  |  |  |
|  | Order 4 | 428.584 | 2 | 214.292 |  |  |  |  |  |
|  | Order 5 | 101.223 | 2 | 50.611 |  |  |  |  |  |
|  | Order 6 | 20.960 | 2 | 10.480 |  |  |  |  |  |
|  | Order 7 | 279.608 | 2 | 139.804 |  |  |  |  |  |
|  | Order 8 | 44.128 | 2 | 22.064 |  |  |  |  |  |
|  | Order 9 | 94.483 | 2 | 47.241 |  |  |  |  |  |
|  | Order 10 | 282.310 | 2 | 141.155 |  |  |  |  |  |
|  | Order 11 | 2.858 | 2 | 1.429 |  |  |  |  |  |
| a. Computed using alpha = .05 | | | | | | | | | |

| **Tests of Between-Subjects Effects** | | | | | | | | |
| --- | --- | --- | --- | --- | --- | --- | --- | --- |
| Measure: MEASURE_1  Transformed Variable: Average | | | | | | | | |
| Source | Type III Sum of Squares | df | Mean Square | F | Sig. | Partial Eta Squared | Noncent. Parameter | Observed Power^a^ |
| Intercept | 90777.852 | 1 | 90777.852 | 27.764 | .034 | .933 | 27.764 | .755 |
| Error | 6539.292 | 2 | 3269.646 |  |  |  |  |  |
| a. Computed using alpha = .05 | | | | | | | | |

**Estimated Marginal Means**

**Round**

| **Estimates** | | | | |
| --- | --- | --- | --- | --- |
| Measure: MEASURE_1 | | | | |
| Round | Mean | Std. Error | 95% Confidence Interval | |
|  |  |  | Lower Bound | Upper Bound |
| 1 | 51.825 | 5.200 | 29.454 | 74.197 |
| 2 | 52.643 | 14.192 | -8.418 | 113.704 |
| 3 | 80.881 | 4.237 | 62.652 | 99.110 |
| 4 | 43.117 | 14.444 | -19.031 | 105.265 |
| 5 | 50.727 | 7.453 | 18.657 | 82.796 |
| 6 | 63.726 | 9.143 | 24.388 | 103.064 |
| 7 | 50.585 | 7.637 | 17.725 | 83.445 |
| 8 | 34.194 | 4.183 | 16.194 | 52.193 |
| 9 | 39.458 | 12.986 | -16.417 | 95.334 |
| 10 | 43.086 | 15.244 | -22.502 | 108.673 |
| 11 | 50.553 | 13.551 | -7.750 | 108.856 |
| 12 | 41.793 | 8.382 | 5.728 | 77.857 |

| **Pairwise Comparisons** | | | | | | |
| --- | --- | --- | --- | --- | --- | --- |
| Measure: MEASURE_1 | | | | | | |
| (I) Round | (J) Round | Mean Difference (I-J) | Std. Error | Sig.^b^ | 95% Confidence Interval for Difference^b^ | |
|  |  |  |  |  | Lower Bound | Upper Bound |
| 1 | 2 | -.817 | 12.287 | 1.000 | -446.962 | 445.327 |
|  | 3 | -29.055 | 4.486 | 1.000 | -191.956 | 133.846 |
|  | 4 | 8.708 | 11.222 | 1.000 | -398.786 | 416.203 |
|  | 5 | 1.099 | 5.481 | 1.000 | -197.937 | 200.135 |
|  | 6 | -11.901 | 7.286 | 1.000 | -276.468 | 252.666 |
|  | 7 | 1.241 | 5.264 | 1.000 | -189.912 | 192.393 |
|  | 8 | 17.632 | 4.240 | 1.000 | -136.314 | 171.577 |
|  | 9 | 12.367 | 10.850 | 1.000 | -381.619 | 406.353 |
|  | 10 | 8.740 | 12.427 | 1.000 | -442.509 | 459.988 |
|  | 11 | 1.272 | 10.226 | 1.000 | -370.035 | 372.579 |
|  | 12 | 10.033 | 6.325 | 1.000 | -219.647 | 239.713 |
| 2 | 1 | .817 | 12.287 | 1.000 | -445.327 | 446.962 |
|  | 3 | -28.238 | 9.961 | 1.000 | -389.950 | 333.474 |
|  | 4 | 9.526 | 3.812 | 1.000 | -128.884 | 147.935 |
|  | 5 | 1.916 | 7.021 | 1.000 | -253.036 | 256.868 |
|  | 6 | -11.083 | 5.169 | 1.000 | -198.775 | 176.608 |
|  | 7 | 2.058 | 7.087 | 1.000 | -255.279 | 259.395 |
|  | 8 | 18.449 | 10.040 | 1.000 | -346.120 | 383.019 |
|  | 9 | 13.184 | 1.475 | .811 | -40.378 | 66.746 |
|  | 10 | 9.557 | 2.724 | 1.000 | -89.347 | 108.461 |
|  | 11 | 2.090 | 4.190 | 1.000 | -150.064 | 154.243 |
|  | 12 | 10.850 | 6.079 | 1.000 | -209.901 | 231.602 |
| 3 | 1 | 29.055 | 4.486 | 1.000 | -133.846 | 191.956 |
|  | 2 | 28.238 | 9.961 | 1.000 | -333.474 | 389.950 |
|  | 4 | 37.763 | 10.350 | 1.000 | -338.074 | 413.601 |
|  | 5 | 30.154 | 3.319 | .785 | -90.355 | 150.663 |
|  | 6 | 17.154 | 4.916 | 1.000 | -161.355 | 195.664 |
|  | 7 | 30.296 | 3.609 | .917 | -100.745 | 161.336 |
|  | 8 | 46.687^*^ | .247 | .002 | 37.720 | 55.654 |
|  | 9 | 41.422 | 8.750 | 1.000 | -276.313 | 359.158 |
|  | 10 | 37.795 | 11.052 | 1.000 | -363.514 | 439.103 |
|  | 11 | 30.327 | 9.508 | 1.000 | -314.915 | 375.569 |
|  | 12 | 39.088 | 4.204 | .750 | -113.561 | 191.737 |
| 4 | 1 | -8.708 | 11.222 | 1.000 | -416.203 | 398.786 |
|  | 2 | -9.526 | 3.812 | 1.000 | -147.935 | 128.884 |
|  | 3 | -37.763 | 10.350 | 1.000 | -413.601 | 338.074 |
|  | 5 | -7.609 | 7.033 | 1.000 | -262.983 | 247.764 |
|  | 6 | -20.609 | 5.646 | 1.000 | -225.607 | 184.389 |
|  | 7 | -7.468 | 6.807 | 1.000 | -254.650 | 239.715 |
|  | 8 | 8.924 | 10.338 | 1.000 | -366.455 | 384.303 |
|  | 9 | 3.659 | 3.145 | 1.000 | -110.523 | 117.841 |
|  | 10 | .032 | 1.625 | 1.000 | -58.986 | 59.049 |
|  | 11 | -7.436 | 1.008 | 1.000 | -44.029 | 29.157 |
|  | 12 | 1.325 | 6.165 | 1.000 | -222.544 | 225.194 |
| 5 | 1 | -1.099 | 5.481 | 1.000 | -200.135 | 197.937 |
|  | 2 | -1.916 | 7.021 | 1.000 | -256.868 | 253.036 |
|  | 3 | -30.154 | 3.319 | .785 | -150.663 | 90.355 |
|  | 4 | 7.609 | 7.033 | 1.000 | -247.764 | 262.983 |
|  | 6 | -12.999 | 1.862 | 1.000 | -80.612 | 54.613 |
|  | 7 | .142 | .541 | 1.000 | -19.490 | 19.774 |
|  | 8 | 16.533 | 3.307 | 1.000 | -103.542 | 136.608 |
|  | 9 | 11.268 | 5.678 | 1.000 | -194.891 | 217.428 |
|  | 10 | 7.641 | 7.793 | 1.000 | -275.344 | 290.626 |
|  | 11 | .173 | 6.192 | 1.000 | -224.661 | 225.008 |
|  | 12 | 8.934 | .954 | .740 | -25.712 | 43.580 |
| 6 | 1 | 11.901 | 7.286 | 1.000 | -252.666 | 276.468 |
|  | 2 | 11.083 | 5.169 | 1.000 | -176.608 | 198.775 |
|  | 3 | -17.154 | 4.916 | 1.000 | -195.664 | 161.355 |
|  | 4 | 20.609 | 5.646 | 1.000 | -184.389 | 225.607 |
|  | 5 | 12.999 | 1.862 | 1.000 | -54.613 | 80.612 |
|  | 7 | 13.141 | 2.025 | 1.000 | -60.406 | 86.689 |
|  | 8 | 29.533 | 4.959 | 1.000 | -150.551 | 209.617 |
|  | 9 | 24.268 | 3.862 | 1.000 | -115.967 | 164.503 |
|  | 10 | 20.640 | 6.162 | 1.000 | -203.127 | 244.407 |
|  | 11 | 13.173 | 4.948 | 1.000 | -166.509 | 192.855 |
|  | 12 | 21.934 | .961 | .126 | -12.961 | 56.829 |
| 7 | 1 | -1.241 | 5.264 | 1.000 | -192.393 | 189.912 |
|  | 2 | -2.058 | 7.087 | 1.000 | -259.395 | 255.279 |
|  | 3 | -30.296 | 3.609 | .917 | -161.336 | 100.745 |
|  | 4 | 7.468 | 6.807 | 1.000 | -239.715 | 254.650 |
|  | 5 | -.142 | .541 | 1.000 | -19.774 | 19.490 |
|  | 6 | -13.141 | 2.025 | 1.000 | -86.689 | 60.406 |
|  | 8 | 16.391 | 3.564 | 1.000 | -113.010 | 145.792 |
|  | 9 | 11.126 | 5.696 | 1.000 | -195.717 | 217.970 |
|  | 10 | 7.499 | 7.666 | 1.000 | -270.868 | 285.866 |
|  | 11 | .032 | 5.927 | 1.000 | -215.197 | 215.260 |
|  | 12 | 8.792 | 1.065 | .948 | -29.892 | 47.476 |
| 8 | 1 | -17.632 | 4.240 | 1.000 | -171.577 | 136.314 |
|  | 2 | -18.449 | 10.040 | 1.000 | -383.019 | 346.120 |
|  | 3 | -46.687^*^ | .247 | .002 | -55.654 | -37.720 |
|  | 4 | -8.924 | 10.338 | 1.000 | -384.303 | 366.455 |
|  | 5 | -16.533 | 3.307 | 1.000 | -136.608 | 103.542 |
|  | 6 | -29.533 | 4.959 | 1.000 | -209.617 | 150.551 |
|  | 7 | -16.391 | 3.564 | 1.000 | -145.792 | 113.010 |
|  | 9 | -5.265 | 8.808 | 1.000 | -325.097 | 314.568 |
|  | 10 | -8.892 | 11.073 | 1.000 | -410.964 | 393.180 |
|  | 11 | -16.360 | 9.482 | 1.000 | -360.654 | 327.935 |
|  | 12 | -7.599 | 4.214 | 1.000 | -160.599 | 145.401 |
| 9 | 1 | -12.367 | 10.850 | 1.000 | -406.353 | 381.619 |
|  | 2 | -13.184 | 1.475 | .811 | -66.746 | 40.378 |
|  | 3 | -41.422 | 8.750 | 1.000 | -359.158 | 276.313 |
|  | 4 | -3.659 | 3.145 | 1.000 | -117.841 | 110.523 |
|  | 5 | -11.268 | 5.678 | 1.000 | -217.428 | 194.891 |
|  | 6 | -24.268 | 3.862 | 1.000 | -164.503 | 115.967 |
|  | 7 | -11.126 | 5.696 | 1.000 | -217.970 | 195.717 |
|  | 8 | 5.265 | 8.808 | 1.000 | -314.568 | 325.097 |
|  | 10 | -3.627 | 2.724 | 1.000 | -102.525 | 95.270 |
|  | 11 | -11.095 | 3.188 | 1.000 | -126.852 | 104.663 |
|  | 12 | -2.334 | 4.725 | 1.000 | -173.906 | 169.238 |
| 10 | 1 | -8.740 | 12.427 | 1.000 | -459.988 | 442.509 |
|  | 2 | -9.557 | 2.724 | 1.000 | -108.461 | 89.347 |
|  | 3 | -37.795 | 11.052 | 1.000 | -439.103 | 363.514 |
|  | 4 | -.032 | 1.625 | 1.000 | -59.049 | 58.986 |
|  | 5 | -7.641 | 7.793 | 1.000 | -290.626 | 275.344 |
|  | 6 | -20.640 | 6.162 | 1.000 | -244.407 | 203.127 |
|  | 7 | -7.499 | 7.666 | 1.000 | -285.866 | 270.868 |
|  | 8 | 8.892 | 11.073 | 1.000 | -393.180 | 410.964 |
|  | 9 | 3.627 | 2.724 | 1.000 | -95.270 | 102.525 |
|  | 11 | -7.468 | 2.507 | 1.000 | -98.486 | 83.551 |
|  | 12 | 1.293 | 6.862 | 1.000 | -247.876 | 250.462 |
| 11 | 1 | -1.272 | 10.226 | 1.000 | -372.579 | 370.035 |
|  | 2 | -2.090 | 4.190 | 1.000 | -154.243 | 150.064 |
|  | 3 | -30.327 | 9.508 | 1.000 | -375.569 | 314.915 |
|  | 4 | 7.436 | 1.008 | 1.000 | -29.157 | 44.029 |
|  | 5 | -.173 | 6.192 | 1.000 | -225.008 | 224.661 |
|  | 6 | -13.173 | 4.948 | 1.000 | -192.855 | 166.509 |
|  | 7 | -.032 | 5.927 | 1.000 | -215.260 | 215.197 |
|  | 8 | 16.360 | 9.482 | 1.000 | -327.935 | 360.654 |
|  | 9 | 11.095 | 3.188 | 1.000 | -104.663 | 126.852 |
|  | 10 | 7.468 | 2.507 | 1.000 | -83.551 | 98.486 |
|  | 12 | 8.761 | 5.366 | 1.000 | -186.078 | 203.599 |
| 12 | 1 | -10.033 | 6.325 | 1.000 | -239.713 | 219.647 |
|  | 2 | -10.850 | 6.079 | 1.000 | -231.602 | 209.901 |
|  | 3 | -39.088 | 4.204 | .750 | -191.737 | 113.561 |
|  | 4 | -1.325 | 6.165 | 1.000 | -225.194 | 222.544 |
|  | 5 | -8.934 | .954 | .740 | -43.580 | 25.712 |
|  | 6 | -21.934 | .961 | .126 | -56.829 | 12.961 |
|  | 7 | -8.792 | 1.065 | .948 | -47.476 | 29.892 |
|  | 8 | 7.599 | 4.214 | 1.000 | -145.401 | 160.599 |
|  | 9 | 2.334 | 4.725 | 1.000 | -169.238 | 173.906 |
|  | 10 | -1.293 | 6.862 | 1.000 | -250.462 | 247.876 |
|  | 11 | -8.761 | 5.366 | 1.000 | -203.599 | 186.078 |
| Based on estimated marginal means | | | | | | |
| *. The mean difference is significant at the .05 level. | | | | | | |
| b. Adjustment for multiple comparisons: Bonferroni. | | | | | | |

**General Linear Model**

| **Multivariate Tests** | | | | | | | | | |
| --- | --- | --- | --- | --- | --- | --- | --- | --- | --- |
|  | Value | F | Hypothesis df | Error df | Sig. | Partial Eta Squared | Noncent. Parameter | Observed Power^b^ |  |
| Pillai's trace | .973 | 17.991^a^ | 2.000 | 1.000 | .164 | .973 | 35.982 | .239 |  |
| Wilks' lambda | .027 | 17.991^a^ | 2.000 | 1.000 | .164 | .973 | 35.982 | .239 |  |
| Hotelling's trace | 35.982 | 17.991^a^ | 2.000 | 1.000 | .164 | .973 | 35.982 | .239 |  |
| Roy's largest root | 35.982 | 17.991^a^ | 2.000 | 1.000 | .164 | .973 | 35.982 | .239 |  |
| Each F tests the multivariate effect of Round. These tests are based on the linearly independent pairwise comparisons among the estimated marginal means. | | | | | | | | | |
| a. Exact statistic | | | | | | | | | |
| b. Computed using alpha = .05  **Profile Plots**  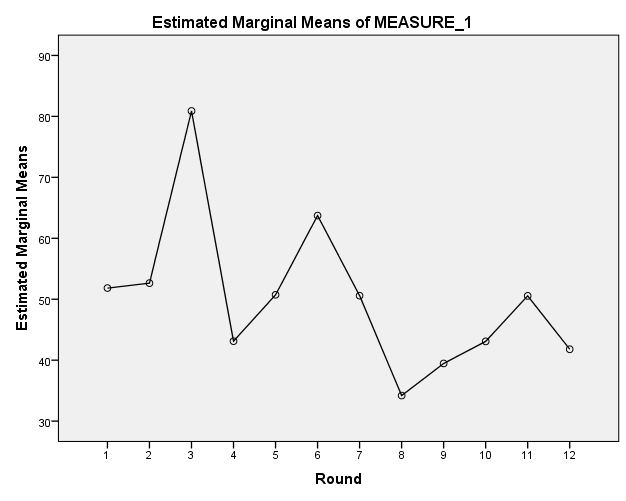 | | | | | | | | | |

**Supplementary Tables 5b. Repeated Measures Analysis of Variance (ANOVA) on the Specific activity of immobilized A.O β-Gal.**

| **Descriptive Statistics** | | | |
| --- | --- | --- | --- |
|  | Mean | Std. Deviation | N |
| Act-R1 | 60.678134 | 6.7569466 | 3 |
| Act-R2 | 71.686206 | 7.3014519 | 3 |
| Act-R3 | 70.566473 | 6.0789520 | 3 |
| Act-R4 | 59.905361 | 1.6815963 | 3 |
| Act-R5 | 62.121169 | 3.8178635 | 3 |
| Act-R6 | 61.103947 | 3.5120932 | 3 |
| Act-R7 | 62.602180 | 11.5891079 | 3 |
| Act-R8 | 66.655296 | 30.3046295 | 3 |
| Act-R9 | 63.319755 | 40.4763381 | 3 |
| Act-R10 | 46.728793 | 9.0560132 | 3 |
| Act-R11 | 60.268091 | 16.5689200 | 3 |
| Act-R12 | 45.916593 | 17.3374379 | 3 |

| **Multivariate Tests^a^** | | | | | | | | | |
| --- | --- | --- | --- | --- | --- | --- | --- | --- | --- |
| Effect | | Value | F | Hypothesis df | Error df | Sig. | Partial Eta Squared | Noncent. Parameter | Observed Power^c^ |
| Round | Pillai's Trace | .^b^ | . | . | . | . | . | . | . |
|  | Wilks' Lambda | .^b^ | . | . | . | . | . | . | . |
|  | Hotelling's Trace | .^b^ | . | . | . | . | . | . | . |
|  | Roy's Largest Root | .^b^ | . | . | . | . | . | . | . |
| a. Design: Intercept  Within Subjects Design: Round | | | | | | | | | |
| b. Cannot produce multivariate test statistics because of insufficient residual degrees of freedom. | | | | | | | | | |
| c. Computed using alpha = .05 | | | | | | | | | |

| **Mauchly's Test of Sphericity^a^** | | | | | | | |
| --- | --- | --- | --- | --- | --- | --- | --- |
| Measure: MEASURE_1 | | | | | | | |
| Within Subjects Effect | Mauchly's W | Approx. Chi-Square | df | Sig. | Epsilon^b^ | | |
|  |  |  |  |  | Greenhouse-Geisser | Huynh-Feldt | Lower-bound |
| Round | .000 | . | 65 | . | .096 | .113 | .091 |
| Tests the null hypothesis that the error covariance matrix of the orthonormalized transformed dependent variables is proportional to an identity matrix. | | | | | | | |
| a. Design: Intercept  Within Subjects Design: Round | | | | | | | |
| b. May be used to adjust the degrees of freedom for the averaged tests of significance. Corrected tests are displayed in the Tests of Within-Subjects Effects table. | | | | | | | |

| **Tests of Within-Subjects Effects** | | | | | | | | | |
| --- | --- | --- | --- | --- | --- | --- | --- | --- | --- |
| Measure: MEASURE_1 | | | | | | | | | |
| Source | | Type III Sum of Squares | df | Mean Square | F | Sig. | Partial Eta Squared | Noncent. Parameter | Observed Power^a^ |
| Round | Sphericity Assumed | 2039.725 | 11 | 185.430 | .857 | .591 | .300 | 9.427 | .333 |
|  | Greenhouse-Geisser | 2039.725 | 1.056 | 1931.185 | .857 | .455 | .300 | .905 | .091 |
|  | Huynh-Feldt | 2039.725 | 1.238 | 1647.328 | .857 | .464 | .300 | 1.061 | .097 |
|  | Lower-bound | 2039.725 | 1.000 | 2039.725 | .857 | .452 | .300 | .857 | .089 |
| Error(Round) | Sphericity Assumed | 4759.974 | 22 | 216.362 |  |  |  |  |  |
|  | Greenhouse-Geisser | 4759.974 | 2.112 | 2253.342 |  |  |  |  |  |
|  | Huynh-Feldt | 4759.974 | 2.476 | 1922.132 |  |  |  |  |  |
|  | Lower-bound | 4759.974 | 2.000 | 2379.987 |  |  |  |  |  |
| a. Computed using alpha = .05 | | | | | | | | | |

| **Tests of Within-Subjects Contrasts** | | | | | | | | | |
| --- | --- | --- | --- | --- | --- | --- | --- | --- | --- |
| Measure: MEASURE_1 | | | | | | | | | |
| Source | Round | Type III Sum of Squares | df | Mean Square | F | Sig. | Partial Eta Squared | Noncent. Parameter | Observed Power^a^ |
| Round | Linear | 838.453 | 1 | 838.453 | .861 | .451 | .301 | .861 | .089 |
|  | Quadratic | 193.493 | 1 | 193.493 | 10.244 | .085 | .837 | 10.244 | .423 |
|  | Cubic | 10.508 | 1 | 10.508 | .017 | .907 | .009 | .017 | .051 |
|  | Order 4 | 96.952 | 1 | 96.952 | 1.597 | .334 | .444 | 1.597 | .121 |
|  | Order 5 | 185.823 | 1 | 185.823 | 1.298 | .373 | .394 | 1.298 | .108 |
|  | Order 6 | 171.270 | 1 | 171.270 | 1.030 | .417 | .340 | 1.030 | .097 |
|  | Order 7 | 209.302 | 1 | 209.302 | 2.160 | .279 | .519 | 2.160 | .145 |
|  | Order 8 | 148.763 | 1 | 148.763 | .754 | .477 | .274 | .754 | .084 |
|  | Order 9 | 178.458 | 1 | 178.458 | 2.282 | .270 | .533 | 2.282 | .150 |
|  | Order 10 | 2.788 | 1 | 2.788 | .097 | .785 | .046 | .097 | .054 |
|  | Order 11 | 3.915 | 1 | 3.915 | .279 | .650 | .122 | .279 | .063 |
| Error(Round) | Linear | 1946.582 | 2 | 973.291 |  |  |  |  |  |
|  | Quadratic | 37.775 | 2 | 18.888 |  |  |  |  |  |
|  | Cubic | 1205.342 | 2 | 602.671 |  |  |  |  |  |
|  | Order 4 | 121.381 | 2 | 60.691 |  |  |  |  |  |
|  | Order 5 | 286.261 | 2 | 143.130 |  |  |  |  |  |
|  | Order 6 | 332.543 | 2 | 166.272 |  |  |  |  |  |
|  | Order 7 | 193.759 | 2 | 96.880 |  |  |  |  |  |
|  | Order 8 | 394.521 | 2 | 197.261 |  |  |  |  |  |
|  | Order 9 | 156.391 | 2 | 78.195 |  |  |  |  |  |
|  | Order 10 | 57.321 | 2 | 28.660 |  |  |  |  |  |
|  | Order 11 | 28.097 | 2 | 14.048 |  |  |  |  |  |
| a. Computed using alpha = .05 | | | | | | | | | |

| **Tests of Between-Subjects Effects** | | | | | | | | |
| --- | --- | --- | --- | --- | --- | --- | --- | --- |
| Measure: MEASURE_1  Transformed Variable: Average | | | | | | | | |
| Source | Type III Sum of Squares | df | Mean Square | F | Sig. | Partial Eta Squared | Noncent. Parameter | Observed Power^a^ |
| Intercept | 133792.081 | 1 | 133792.081 | 118.002 | .008 | .983 | 118.002 | .997 |
| Error | 2267.624 | 2 | 1133.812 |  |  |  |  |  |
| a. Computed using alpha = .05 | | | | | | | | |

**Estimated Marginal Means**

**Round**

| **Estimates** | | | | |
| --- | --- | --- | --- | --- |
| Measure: MEASURE_1 | | | | |
| Round | Mean | Std. Error | 95% Confidence Interval | |
|  |  |  | Lower Bound | Upper Bound |
| 1 | 60.678 | 3.901 | 43.893 | 77.463 |
| 2 | 71.686 | 4.215 | 53.548 | 89.824 |
| 3 | 70.566 | 3.510 | 55.466 | 85.667 |
| 4 | 59.905 | .971 | 55.728 | 64.083 |
| 5 | 62.121 | 2.204 | 52.637 | 71.605 |
| 6 | 61.104 | 2.028 | 52.379 | 69.828 |
| 7 | 62.602 | 6.691 | 33.813 | 91.391 |
| 8 | 66.655 | 17.496 | -8.626 | 141.936 |
| 9 | 63.320 | 23.369 | -37.229 | 163.869 |
| 10 | 46.729 | 5.228 | 24.232 | 69.225 |
| 11 | 60.268 | 9.566 | 19.109 | 101.428 |
| 12 | 45.917 | 10.010 | 2.848 | 88.985 |

| **Pairwise Comparisons** | | | | | | |
| --- | --- | --- | --- | --- | --- | --- |
| Measure: MEASURE_1 | | | | | | |
| (I) Round | (J) Round | Mean Difference (I-J) | Std. Error | Sig.^a^ | 95% Confidence Interval for Difference^a^ | |
|  |  |  |  |  | Lower Bound | Upper Bound |
| 1 | 2 | -11.008 | 8.116 | 1.000 | -305.702 | 283.686 |
|  | 3 | -9.888 | 7.361 | 1.000 | -277.166 | 257.390 |
|  | 4 | .773 | 4.700 | 1.000 | -169.900 | 171.446 |
|  | 5 | -1.443 | 5.642 | 1.000 | -206.310 | 203.424 |
|  | 6 | -.426 | 2.764 | 1.000 | -100.793 | 99.941 |
|  | 7 | -1.924 | 2.797 | 1.000 | -103.472 | 99.624 |
|  | 8 | -5.977 | 13.596 | 1.000 | -499.646 | 487.691 |
|  | 9 | -2.642 | 19.567 | 1.000 | -713.154 | 707.871 |
|  | 10 | 13.949 | 1.674 | .931 | -46.852 | 74.750 |
|  | 11 | .410 | 5.705 | 1.000 | -206.747 | 207.567 |
|  | 12 | 14.762 | 6.116 | 1.000 | -207.327 | 236.850 |
| 2 | 1 | 11.008 | 8.116 | 1.000 | -283.686 | 305.702 |
|  | 3 | 1.120 | 1.227 | 1.000 | -43.422 | 45.661 |
|  | 4 | 11.781 | 3.487 | 1.000 | -114.842 | 138.403 |
|  | 5 | 9.565 | 3.089 | 1.000 | -102.609 | 121.739 |
|  | 6 | 10.582 | 5.902 | 1.000 | -203.744 | 224.909 |
|  | 7 | 9.084 | 10.901 | 1.000 | -386.734 | 404.902 |
|  | 8 | 5.031 | 21.709 | 1.000 | -783.261 | 793.323 |
|  | 9 | 8.366 | 27.486 | 1.000 | -989.677 | 1006.410 |
|  | 10 | 24.957 | 9.368 | 1.000 | -315.210 | 365.124 |
|  | 11 | 11.418 | 13.772 | 1.000 | -488.650 | 511.486 |
|  | 12 | 25.770 | 14.225 | 1.000 | -490.745 | 542.284 |
| 3 | 1 | 9.888 | 7.361 | 1.000 | -257.390 | 277.166 |
|  | 2 | -1.120 | 1.227 | 1.000 | -45.661 | 43.422 |
|  | 4 | 10.661 | 3.008 | 1.000 | -98.554 | 119.876 |
|  | 5 | 8.445 | 3.081 | 1.000 | -103.436 | 120.326 |
|  | 6 | 9.463 | 4.945 | 1.000 | -170.083 | 189.008 |
|  | 7 | 7.964 | 10.157 | 1.000 | -360.848 | 376.776 |
|  | 8 | 3.911 | 20.934 | 1.000 | -756.210 | 764.032 |
|  | 9 | 7.247 | 26.878 | 1.000 | -968.712 | 983.206 |
|  | 10 | 23.838 | 8.738 | 1.000 | -293.454 | 341.129 |
|  | 11 | 10.298 | 12.925 | 1.000 | -459.020 | 479.616 |
|  | 12 | 24.650 | 13.417 | 1.000 | -462.524 | 511.824 |
| 4 | 1 | -.773 | 4.700 | 1.000 | -171.446 | 169.900 |
|  | 2 | -11.781 | 3.487 | 1.000 | -138.403 | 114.842 |
|  | 3 | -10.661 | 3.008 | 1.000 | -119.876 | 98.554 |
|  | 5 | -2.216 | 1.252 | 1.000 | -47.676 | 43.244 |
|  | 6 | -1.199 | 2.997 | 1.000 | -110.025 | 107.628 |
|  | 7 | -2.697 | 7.455 | 1.000 | -273.381 | 267.987 |
|  | 8 | -6.750 | 18.261 | 1.000 | -669.823 | 656.323 |
|  | 9 | -3.414 | 24.001 | 1.000 | -874.910 | 868.081 |
|  | 10 | 13.177 | 5.883 | 1.000 | -200.438 | 226.791 |
|  | 11 | -.363 | 10.401 | 1.000 | -378.039 | 377.314 |
|  | 12 | 13.989 | 10.813 | 1.000 | -378.658 | 406.635 |
| 5 | 1 | 1.443 | 5.642 | 1.000 | -203.424 | 206.310 |
|  | 2 | -9.565 | 3.089 | 1.000 | -121.739 | 102.609 |
|  | 3 | -8.445 | 3.081 | 1.000 | -120.326 | 103.436 |
|  | 4 | 2.216 | 1.252 | 1.000 | -43.244 | 47.676 |
|  | 6 | 1.017 | 4.229 | 1.000 | -152.530 | 154.565 |
|  | 7 | -.481 | 8.304 | 1.000 | -302.008 | 301.046 |
|  | 8 | -4.534 | 19.056 | 1.000 | -696.492 | 687.423 |
|  | 9 | -1.199 | 24.586 | 1.000 | -893.929 | 891.532 |
|  | 10 | 15.392 | 6.616 | 1.000 | -224.836 | 255.621 |
|  | 11 | 1.853 | 11.332 | 1.000 | -409.624 | 413.330 |
|  | 12 | 16.205 | 11.694 | 1.000 | -408.420 | 440.829 |
| 6 | 1 | .426 | 2.764 | 1.000 | -99.941 | 100.793 |
|  | 2 | -10.582 | 5.902 | 1.000 | -224.909 | 203.744 |
|  | 3 | -9.463 | 4.945 | 1.000 | -189.008 | 170.083 |
|  | 4 | 1.199 | 2.997 | 1.000 | -107.628 | 110.025 |
|  | 5 | -1.017 | 4.229 | 1.000 | -154.565 | 152.530 |
|  | 7 | -1.498 | 5.435 | 1.000 | -198.850 | 195.854 |
|  | 8 | -5.551 | 16.071 | 1.000 | -589.109 | 578.007 |
|  | 9 | -2.216 | 22.244 | 1.000 | -809.918 | 805.486 |
|  | 10 | 14.375 | 4.403 | 1.000 | -145.515 | 174.265 |
|  | 11 | .836 | 8.015 | 1.000 | -290.210 | 291.881 |
|  | 12 | 15.187 | 8.545 | 1.000 | -295.082 | 325.457 |
| 7 | 1 | 1.924 | 2.797 | 1.000 | -99.624 | 103.472 |
|  | 2 | -9.084 | 10.901 | 1.000 | -404.902 | 386.734 |
|  | 3 | -7.964 | 10.157 | 1.000 | -376.776 | 360.848 |
|  | 4 | 2.697 | 7.455 | 1.000 | -267.987 | 273.381 |
|  | 5 | .481 | 8.304 | 1.000 | -301.046 | 302.008 |
|  | 6 | 1.498 | 5.435 | 1.000 | -195.854 | 198.850 |
|  | 8 | -4.053 | 10.810 | 1.000 | -396.566 | 388.460 |
|  | 9 | -.718 | 16.811 | 1.000 | -611.131 | 609.696 |
|  | 10 | 15.873 | 1.838 | .867 | -50.865 | 82.611 |
|  | 11 | 2.334 | 3.111 | 1.000 | -110.622 | 115.291 |
|  | 12 | 16.686 | 3.394 | 1.000 | -106.553 | 139.924 |
| 8 | 1 | 5.977 | 13.596 | 1.000 | -487.691 | 499.646 |
|  | 2 | -5.031 | 21.709 | 1.000 | -793.323 | 783.261 |
|  | 3 | -3.911 | 20.934 | 1.000 | -764.032 | 756.210 |
|  | 4 | 6.750 | 18.261 | 1.000 | -656.323 | 669.823 |
|  | 5 | 4.534 | 19.056 | 1.000 | -687.423 | 696.492 |
|  | 6 | 5.551 | 16.071 | 1.000 | -578.007 | 589.109 |
|  | 7 | 4.053 | 10.810 | 1.000 | -388.460 | 396.566 |
|  | 9 | 3.336 | 7.091 | 1.000 | -254.148 | 260.819 |
|  | 10 | 19.927 | 12.441 | 1.000 | -431.820 | 471.673 |
|  | 11 | 6.387 | 8.082 | 1.000 | -287.071 | 299.846 |
|  | 12 | 20.739 | 7.527 | 1.000 | -252.566 | 294.043 |
| 9 | 1 | 2.642 | 19.567 | 1.000 | -707.871 | 713.154 |
|  | 2 | -8.366 | 27.486 | 1.000 | -1006.410 | 989.677 |
|  | 3 | -7.247 | 26.878 | 1.000 | -983.206 | 968.712 |
|  | 4 | 3.414 | 24.001 | 1.000 | -868.081 | 874.910 |
|  | 5 | 1.199 | 24.586 | 1.000 | -891.532 | 893.929 |
|  | 6 | 2.216 | 22.244 | 1.000 | -805.486 | 809.918 |
|  | 7 | .718 | 16.811 | 1.000 | -609.696 | 611.131 |
|  | 8 | -3.336 | 7.091 | 1.000 | -260.819 | 254.148 |
|  | 10 | 16.591 | 18.142 | 1.000 | -642.161 | 675.343 |
|  | 11 | 3.052 | 14.589 | 1.000 | -526.695 | 532.798 |
|  | 12 | 17.403 | 13.916 | 1.000 | -487.909 | 522.715 |
| 10 | 1 | -13.949 | 1.674 | .931 | -74.750 | 46.852 |
|  | 2 | -24.957 | 9.368 | 1.000 | -365.124 | 315.210 |
|  | 3 | -23.838 | 8.738 | 1.000 | -341.129 | 293.454 |
|  | 4 | -13.177 | 5.883 | 1.000 | -226.791 | 200.438 |
|  | 5 | -15.392 | 6.616 | 1.000 | -255.621 | 224.836 |
|  | 6 | -14.375 | 4.403 | 1.000 | -174.265 | 145.515 |
|  | 7 | -15.873 | 1.838 | .867 | -82.611 | 50.865 |
|  | 8 | -19.927 | 12.441 | 1.000 | -471.673 | 431.820 |
|  | 9 | -16.591 | 18.142 | 1.000 | -675.343 | 642.161 |
|  | 11 | -13.539 | 4.944 | 1.000 | -193.066 | 165.987 |
|  | 12 | .812 | 5.177 | 1.000 | -187.174 | 188.799 |
| 11 | 1 | -.410 | 5.705 | 1.000 | -207.567 | 206.747 |
|  | 2 | -11.418 | 13.772 | 1.000 | -511.486 | 488.650 |
|  | 3 | -10.298 | 12.925 | 1.000 | -479.616 | 459.020 |
|  | 4 | .363 | 10.401 | 1.000 | -377.314 | 378.039 |
|  | 5 | -1.853 | 11.332 | 1.000 | -413.330 | 409.624 |
|  | 6 | -.836 | 8.015 | 1.000 | -291.881 | 290.210 |
|  | 7 | -2.334 | 3.111 | 1.000 | -115.291 | 110.622 |
|  | 8 | -6.387 | 8.082 | 1.000 | -299.846 | 287.071 |
|  | 9 | -3.052 | 14.589 | 1.000 | -532.798 | 526.695 |
|  | 10 | 13.539 | 4.944 | 1.000 | -165.987 | 193.066 |
|  | 12 | 14.351 | .750 | .179 | -12.877 | 41.580 |
| 12 | 1 | -14.762 | 6.116 | 1.000 | -236.850 | 207.327 |
|  | 2 | -25.770 | 14.225 | 1.000 | -542.284 | 490.745 |
|  | 3 | -24.650 | 13.417 | 1.000 | -511.824 | 462.524 |
|  | 4 | -13.989 | 10.813 | 1.000 | -406.635 | 378.658 |
|  | 5 | -16.205 | 11.694 | 1.000 | -440.829 | 408.420 |
|  | 6 | -15.187 | 8.545 | 1.000 | -325.457 | 295.082 |
|  | 7 | -16.686 | 3.394 | 1.000 | -139.924 | 106.553 |
|  | 8 | -20.739 | 7.527 | 1.000 | -294.043 | 252.566 |
|  | 9 | -17.403 | 13.916 | 1.000 | -522.715 | 487.909 |
|  | 10 | -.812 | 5.177 | 1.000 | -188.799 | 187.174 |
|  | 11 | -14.351 | .750 | .179 | -41.580 | 12.877 |
| Based on estimated marginal means | | | | | | |
| a. Adjustment for multiple comparisons: Bonferroni. | | | | | | |

| **Multivariate Tests** | | | | | | | | |
| --- | --- | --- | --- | --- | --- | --- | --- | --- |
|  | Value | F | Hypothesis df | Error df | Sig. | Partial Eta Squared | Noncent. Parameter | Observed Power^b^ |
| Pillai's trace | .479 | .460^a^ | 2.000 | 1.000 | .722 | .479 | .920 | .061 |
| Wilks' lambda | .521 | .460^a^ | 2.000 | 1.000 | .722 | .479 | .920 | .061 |
| Hotelling's trace | .920 | .460^a^ | 2.000 | 1.000 | .722 | .479 | .920 | .061 |
| Roy's largest root | .920 | .460^a^ | 2.000 | 1.000 | .722 | .479 | .920 | .061 |
| Each F tests the multivariate effect of Round. These tests are based on the linearly independent pairwise comparisons among the estimated marginal means. | | | | | | | | |
| a. Exact statistic | | | | | | | | |
| b. Computed using alpha = .05 | | | | | | | | |
